# Inferring influenza global transmission networks without complete phylogenetic information

**DOI:** 10.1111/eva.12138

**Published:** 2014-01-02

**Authors:** Stéphane Aris-Brosou

**Affiliations:** 1 Department of Biology, Center for Advanced Research in Environmental Genomics, University of OttawaOttawa, ON, Canada; 2 Department of Mathematics and Statistics, University of OttawaOttawa, ON, Canada

**Keywords:** genetic diversity, global dynamics, influenza, PageRank algorithm, prevalence, transmission network

## Abstract

Influenza is one of the most severe respiratory infections affecting humans throughout the world, yet the dynamics of its global transmission network are still contentious. Here, I describe a novel combination of phylogenetics, time series, and graph theory to analyze 14.25 years of data stratified in space and in time, focusing on the main target of the human immune response, the hemagglutinin gene. While bypassing the complete phylogenetic inference of huge data sets, the method still extracts information suggesting that waves of genetic or of nucleotide diversity circulate continuously around the globe for subtypes that undergo sustained transmission over several seasons, such as H3N2 and pandemic H1N1/09, while diversity of prepandemic H1N1 viruses had until 2009 a noncontinuous transmission pattern consistent with a source/sink model. Irrespective of the shift in the structure of H1N1 diversity circulation with the emergence of the pandemic H1N1/09 strain, US prevalence peaks during the winter months when genetic diversity is at its lowest. This suggests that a dominant strain is generally responsible for epidemics and that monitoring genetic and/or nucleotide diversity in real time could provide public health agencies with an indirect estimate of prevalence.

## Introduction

With three to five million cases of acute illness every year leading to 250 000 to 500 000 deaths, influenza is considered to be one of the most severe respiratory infections affecting humans (Rambaut et al. 2008; World Health Organization 2009). The recent history of this infection is, however, punctuated by pandemics that signal, most of the time, the replacement of a viral subtype by a new one: H1N1 first emerged in 1918 during the ‘Spanish influenza’ and was replaced in 1957 by H2N2 with the ‘Asian influenza’, itself superseded in 1968 by H3N2 during the ‘Hong Kong influenza’ (Neumann et al. 2009). The 1977 ‘Russian influenza’ outbreak marked the reemergence of H1N1, but it did not replace the dominant H3N2 and the two subtypes have been cocirculating ever since. The H1N1 2009 ‘Swine influenza’ pandemic did not change the situation, as H3N2 is, to date, still the cause of most infections in humans during each season (Center for Disease Control 2012).

Each subtype indeed shows a seasonal pattern of infection that peaks around week ten in the Northern Hemisphere (Center for Disease Control 2012) and 6 months later in the Southern Hemisphere (Nelson and Holmes 2007). Evidence shows that, on average, these two epidemics are seeded in a tropical region (Rambaut et al. 2008), most likely centered around East and South-East Asia (Russell et al. 2008). On a year-to-year basis, the exact seeding location may vary (Bahl et al. 2011), but data suggest that some strains circulate around the globe and persist for multiple years (Bedford et al. 2010; Bahl et al. 2011). As a result, it is still unclear how this circulation pattern relates to the global dynamics of viral diversity, and in turn how this diversity impacts the health of human populations.

Here, to address these two outstanding questions, I focus on one single protein-coding gene of the influenza virus, the hemagglutinin (HA) gene. This gene was chosen as it is the surface antigen of the influenza virus that is mostly targeted by the host immune response, so that its evolution is expected to reflect the interaction with its human host (Russell et al. 2008). As a consequence, HA is the most sequenced gene among the eight single strand negative RNA segments constituting the influenza A genome: As of October 28, 2013, the Influenza Virus Resource (IVR) (Bao et al. 2008) contains 34 540 sequences (in humans, worldwide, all subtypes confounded), while the neuraminidase (NA) gene, which encodes the second surface antigen of the influenza virus, comes as a distant second in the database with 22 817 sequences (34% fewer sequences). These HA sequences were then analyzed for both H1N1 and H3N2 subtypes. For this, I describe a novel combination of phylogenetic, time series, and network analyses under a stratified design. This approach reveals the existence of waves of genetic diversity that continuously circulate around the world, seasonally, and unveils a potential shift in the transmission pattern of H1N1 at the emergence of the 2009 pandemic. Regardless of this shift, the results show that the proportion of infected people in a given population (prevalence) is at its peak when influenza diversity is at its lowest, at least in the US population.

## Materials and methods

### Data retrieval

Human HA nucleotide sequences of influenza A viruses of subtypes H1N1 and H3N2 were downloaded from the IVR for five World Health Organization (WHO) geographic regions (Asia, Europe, North America, Oceania, and South America—too few sequences [H1: 258; H3: 277] were deposited for Africa) for viruses collected between January 1, 1996 and April 1, 2011. These data were split by quarter for each of the WHO regions. The length of this time window (quarter) was chosen to ensure that most alignments had ≫2 sequences. All data were downloaded in May 2011, resulting in at most 570 data sets (4 quarters for 14.25 years in 5 regions and 2 subtypes). Sequences without information about collection month were discarded. This resulted in 8748 H1 and 6587 H3 sequences. Figure S1 shows the countries from which data were retrieved. Data distribution is depicted in Figure S2, with a more detailed breakdown by region in Figure S3.

In parallel, weekly prevalence data for the USA were collected from the Centers for Disease Control and Prevention (CDC) at http://www.cdc.gov/flu/weekly from the last quarter of 1997 to April 2011. Weekly data were averaged by quarters, as summarized in Figure S4.

### Phylogenetic analyses

Each of the 570 data sets was aligned with Muscle (Edgar 2004) with default parameters. Sequences were not trimmed (Talavera and Castresana 2007; Capella-Gutiérrez et al. 2009) to conserve as many variable sites as possible upstream and downstream of the coding sequence. Because of the potential presence of noncoding sequences in the data and of out-of-frame data, alignments were performed directly on DNA instead of protein sequences (Aris-Brosou 2010; Abdussamad and Aris-Brosou 2011). This was not problematic here as sequences within a quarter and a given region showed high levels of similarity. Alignments were visually inspected with JalView (Waterhouse et al. 2009), misaligned sequences were removed (H1: HM625636, CY083655; H3: FJ769860, EU835537, EU642547, EU642548), and gaps were adjusted manually. Phylogenetic trees for each of these data sets were then estimated using maximum likelihood with FastTree, version 2.1.3 (Price et al. 2010), under the GTR + Γ substitution model, which is general enough to accommodate substitution patterns in those closely related sequences. Only data sets with more than two sequences were analyzed. One hundred bootstrap replicates were generated for each data set with seqboot (Felsenstein 2005) and analyzed with FastTree as above. Tree lengths were computed for each estimated tree by taking the sum of their branch length and were standardized by dividing each of them by the number of sequences. This standardized tree length, used as a measure of genetic diversity, is henceforth denoted *ν*.

As this measure can be sensitive to phylogenetic uncertainty, nucleotide diversity (Nei and Li 1979) *π* was also computed: It is the sum of pairwise distances of *n* aligned sequences, normalized by the number of comparisons *n* (*n*–1)/2; the distance used is the ‘raw’ distance, that is, the (uncorrected) number of pairwise differences. The R package pegas (Paradis 2010) was modified to take care of sums with missing data as pairwise deletion was used to handle gaps.

### Time series and network analyses

Time series analysis was performed at the level of diversity data (*ν* and *π*). To extract seasonality patterns, a simple additive decomposition was performed with the following model (here described for *ν*, but a similar equation was used for *π*):


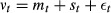
(1)

where, *ν*_*t*_ represents the estimated quarterly values for *ν*, such that for *Q* quarters {*ν*_*t*_: *t* = 1,2,…,*Q*} = {*ν*_1_,*ν*_2_,…,*ν*_*Q*_}. The terms in the right-hand side of equation (1) represent the trend (*m*_*t*_), the seasonal effect (*s*_*t*_), and an error term (*ε*_*t*_) that is generally a sequence of uncorrelated random variables with a mean of zero. The trend *m*_*t*_ was estimated with a moving average centered on *ν*_*t*_. The quarterly additive effect was then estimated as:


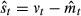
(2)

**Figure 1:**
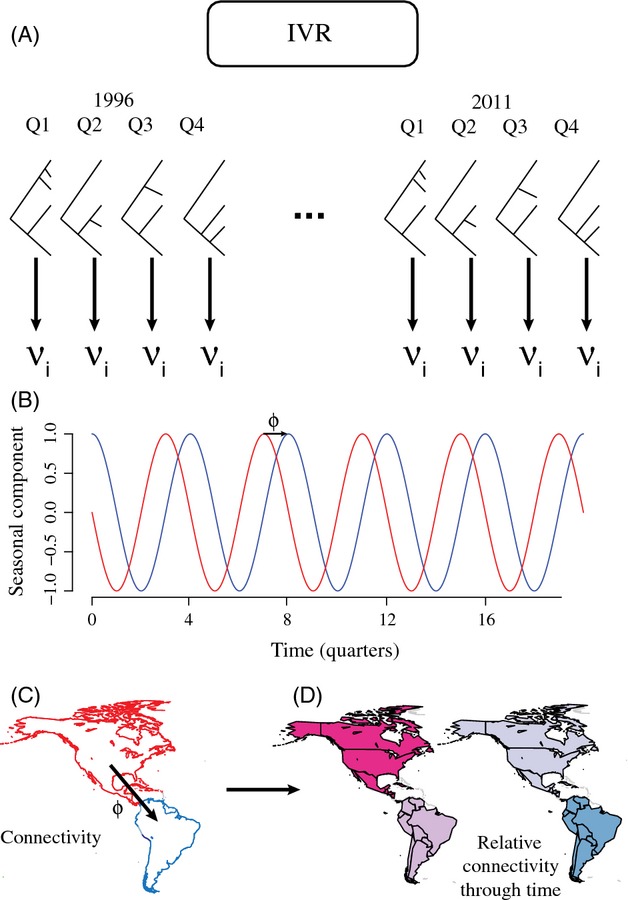
The analytic framework adopted in this study. (A) HA sequences were downloaded from the Influenza Virus Resource (IVR), for both H1 and H3 viruses and stratified by quarters. For each quarter, a phylogenetic tree was estimated by maximum likelihood and used to derive the estimator *ν* of genetic diversity. (B) The detrended seasonal component of *ν* was then extracted for each subtype, within each region (e.g., region R1 in red and R2 in blue). (C) Phase shifts *ϕ* were measured and tested through an autocorrelation analysis. (D) The results were mapped in terms of absolute connectivities and in terms of relative connectivities through time.

The error term is then calculated as in equation (1) (for more details, see Cowpertwait and Metcalfe 2009, pp. 19–22). All time series analyses were performed in R and were based on the stats package (R Development Core Team 2011). The seasonality component of these time series data was extracted as detailed in the electronic supplementary material. The complete design, summarized in Fig. 1, leads to the reconstruction of connectivity networks in terms of either genetic diversity *ν* or nucleotide diversity *π*. The complete analyses were based on both summary statistics.

To compare the reconstructed networks, PageRank eigenvalues (Brin and Page 1998) were computed with R's igraph library (Csardi and Nepusz 2006). All three lags (Q0, Q1, and Q2) were included. Sampling variances of both their maximum and variance across all five WHO regions were obtained by rewiring the networks 10^5^ times.

## Results and discussion

### Seasonality patterns of genetic diversity

HA sequences were split or *stratified* by quarter, region, and subtype to compute an estimate of genetic diversity at these different levels, hereby leading to the analysis of 135 H1 and 201 H3 data sets. The estimate of genetic diversity, first evaluated here, denoted *ν*, is the average tree length of the phylogeny reconstructed for each data set, scaled by the number of sequences entering each data set. This scaling ensures that *ν* is independent of sample size (*t *=* *−0.12, *P* = 0.9052). As such, *ν* represents the average number of substitutions per site per sequence during a given year in a given WHO region. The stratified design has the advantage of being simple to implement, and *ν* is a simple statistic to compute with minimal assumptions. Critically, *ν* does not assume that all sequences in a given quarter in a given WHO region come from the same population: Different populations will be separated by long branch lengths, leading to high estimates of *ν*, while no population structure will lead to small estimates of *ν*. Because the complete analysis only relies on slices in time and in space, it may be expected to be more robust than a full modeling approach based on spatial processes (Lemey et al. 2009) that are potentially time inhomogeneous (Aris-Brosou and Rodrigue 2012). The test of this robustness hypothesis is left to future work.

Because the general approach relies on phylogenetic trees (Fig. 1), the estimates of *ν* might be sensitive to phylogenetic uncertainty. Two additional analyses were taken to address this point. First, average confidence in the phylogenetic trees was estimated by computing mean bootstrap values 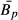 over all the bipartitions (all trees, regions and subtypes confounded). This mean was very close to 70% (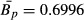), a value showed by Hillis and Bull (1993) to correspond to a 95% probability of bipartitions to be true, and 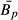 was close to the mode of the distribution of individual bootstrap values (Figure S5).

Alternatively, nucleotide diversity *π*, a measure independent of phylogenetic information, was also computed. In spite of losing some information when compared to *ν* (in the same way that distance methods are less efficient than maximum likelihood at reconstructing trees; e.g., Hasegawa et al. 1991), nucleotide diversity *π* was highly correlated with *ν* (Figure S6). Furthermore, depending on the data sets analyzed, correlations vary between 45% and 96% and are consequently quite high, while the part of the total variance explained by the model fit varies between 39% and 63% (Figure S6). While the analyses below were performed on both *ν* and *π*, results show that *ν* or *π* can be used almost interchangeably without affecting the conclusions.

As with any phylogenetic analysis, the approach described here may be sensitive to sampling biases, where a number of sequences deposited in the database come from a small number of places. This sampling bias is expected to be mitigated by pooling individual locations into WHO regions. Indeed, it is clear from Figure S7 that the five regions are not homogeneously sampled: For instance, data from South America are relatively sparse (see also Figures S2–S3). In spite of this uneven sampling, however, the diversity peaks are uncorrelated (at the *α* = 1% level) at two levels: (i) among the five regions and (ii) between the two subtypes (Table S1). Regardless of this lack of exact spatiotemporal correlation, can one find patterns in each region for each subtype that might be correlated after a time (phase) shift or lag?

To address this question, I extracted the seasonal signal through time, first from the diversity *ν* data. The actual decompositions, shown in Figures S8–S12, reveal that a seasonal signal exists in each region and for each subtype. Figure S13 summarizes these results. A number of patterns emerge from this seasonal decomposition. First, only in Asia are the H1 and H3 diversities peaking at the same time, during Q4. In the other four regions, the peak diversities are asynchronous among subtypes, with H3 diversity generally peaking before H1s, except in Europe where it is the opposite pattern. This general result is consistent with previous evidence showing that H3 viruses exhibit highest diversity early during the epidemic period in the state of New York (Creanza et al. 2010). This first point also suggests that each subtype has its own global dynamics, which may become synchronized in the tropics, probably because tropical regions have more sustained biphasic epidemics per year than other regions (Tamerius et al. 2011). Second, only Oceania exhibits a clear biphasic pattern for *both* subtypes, with a peak in Q3 and a second one in Q1. This pattern would be more expected from a tropical region that sees up to two epidemics per year. Of note, Asia has such a biphasic pattern only for H1 viruses, whose diversity peaks in Q4 and Q2. Third, epidemics in the northern hemisphere (Europe and North America) are generally characterized by single peaks of diversity for both subtypes. On the other hand, Asia, Oceania, and South America show two peaks for H1 viruses, with a 6-month shift for the second peak. Again, this result is expected for tropical regions (Asia and, to some extent, the northern part of Oceania; Fig. 1), which are known to be seeding a new epidemic in each hemisphere with a 6-month shift (Nelson and Holmes 2007). As no H1 sequences were documented in South America prior to the H1N1/09 pandemic (Figure S7), the biphasic H1 diversity in this region is likely a reflection of the biphasic pandemic that occurred in 2009, which is reminiscent of the biphasic pandemic diversity observed in the USA (Nelson et al. 2011) or in Scotland (Lycett et al. 2012).

To assess the general impact of the pandemic H1N1/09 sequences on the results, the seasonality decompositions were conservatively limited to 1996-Q4/2008 (fourth quarter of 2008). While pandemic sequences could have diverged as early as 2006 (Abdussamad and Aris-Brosou 2011), the first casualties were recorded back in March 2009 (Smith et al. 2009). Figure S14 confirms the patterns described above for the whole 14.25-year period. Yet, this similarity does not mean that the detailed correlation structure in terms of genetic diversity *ν*, and hence, global transmission patterns, did not change.

### Structure and stability of global transmission patterns

To better understand the relationship between these peak diversities across both geographic regions and subtypes, a spatiotemporal autocorrelation analysis was performed. In the first step, the autocorrelation functions were computed for H1 and H3 viruses among regions (Figures S15–S17). Then, significant autocorrelations at the 99% level were plotted on a map of the world for three lags: 0, 1, and 2 quarters (Figure S18). For instance, a lag of 0 means that the diversities are synchronized between two regions, while a lag of two quarters indicates a correlation with a 6-month shift, which corresponds to the time difference between seasons in each hemisphere. In the context of global circulation of influenza viruses (Rambaut et al. 2008; Russell et al. 2008), this map represents the temporal connectivity in terms of genetic diversity of these viruses among different regions. Thus, these results naturally lend themselves to graph theory (see, Kaiser 2011).

**Figure 2:**
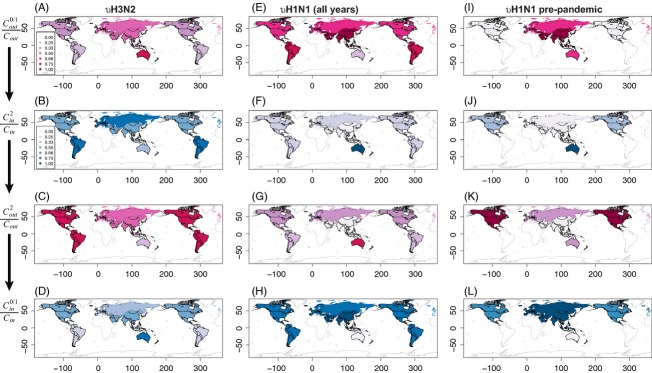
Global relative connectivity of influenza diversity *ν* through time. Each column represents the relative connectivity values for H3N2 (A–D), H1N1 (E–H), and prepandemic H1N1 (I–L) viruses. Each row represents the temporal connection as described in the text. Relative connectivity values are plotted on two scales: out connectivities are in warm colors, while in connectivities are in cold colors [see insets in panels (A) and (B) for scales].

**Figure 3:**
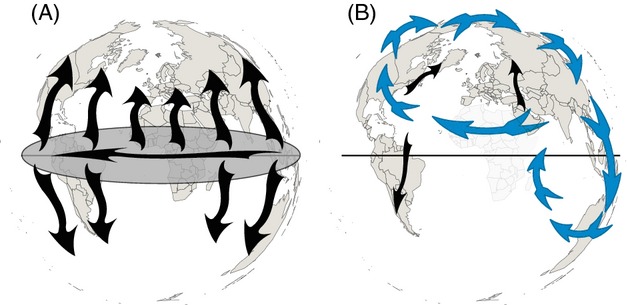
Source/sink and circuit models describing global influenza transmission. (A) In the traditional source/sink model, viral strains emanate from a large source population, assumed to be located between the tropics (ellipsoid around the equator), to seed epidemics in each hemisphere (vertical black arrows), which play the role of viral sinks, from which viruses do not reemerge; strains are replaced through time in the source population (Rambaut et al. 2008). (B) The model proposed here to describe the spread of genetic diversity with a continuous circulation model (blue arrows); outgrowths from the global circuit (black arrows) lead to sink populations. Africa is represented in light gray as no data were available.

H3N2 viruses show two highly connected subnetworks where diversity peaks are synchronous: (i) the Eurasian–Oceania axis (Europe + Asia + Oceania: EAO) and (ii) the American axis (North and South America: NSA; Figure S18A). Diversity peaks during Q4 in EAO and a quarter later in NSA (green arrows in Figure S18A; see also Figure S13). Although each subnetwork represents preferential transmission routes, already known in the case of EAO (Lemey et al. 2009), the connectivities of their nodes are quite distinct. Four ratios are used to reconstruct the connectivity patterns and their temporal dynamics (Fig. 2). Source or distributor nodes, from which diversity emerges, have a high proportion of outgoing edges, between 0 and 1 quarter lags. This leads to the definition of the first two ratios that quantify relative outgoing connectivities at two different lags: at zero or one quarter, 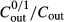 (where 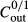 is the outgoing connectivity at lag 0 and 1, and *C*_out_ is the total outgoing connectivity at a particular node) and a half year later, 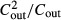, which corresponds to the season shift across hemispheres. Two similar ratios are defined for incoming connections, hereby identifying nodes that receive diversity waves (local sinks). Although with only five nodes, the five WHO regions, assessment of statistical support is limited, a clear pattern emerges. Figure 2A (see Table S2 for actual values) shows that Oceania is the primary immediate distributor of H3N2 diversity, as it has the largest corresponding ratio 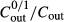
 = 0.75. Two quarters later, South America becomes the main collector node with 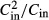
 = 0.75 (Fig. 2B). The remaining diversity is absorbed by Europe and North America. At the same time, South and North America become the main distributors (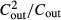
 = 0.75 and 0.67, respectively; Fig. 2C), hereby sustaining the diversity dynamics, while two quarters later Oceania and Europe are now the main collectors (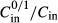
 = 0.67; Fig. 2D). As a result, the diversity of H3N2 viruses does not seem to follow a pure source/sink model (Fig. 3A), but rather acts as a wave rolling across the globe (Fig. 3B): The outgoing connectivity pattern in Fig. 2A matches the incoming pattern observed in Fig. 2D, hereby ‘closing the loop’ through Oceania. This connectivity network, based on the seasonality of diversity peaks, closely mirrors the known average pattern of global spread of H3N2 viruses (Rambaut et al. 2008). This parallel between diversity and ancestry thereby suggests that the assumption that these waves of diversity result from waves of circulating viruses is valid. However, because viral circulation goes around the globe in a year, the notions of starting points and finishing lines fade, and Oceania merely becomes one of the many spatial positions placed on the circuit. The circulation model proposed here for H3N2 (Fig. 3B) is therefore distinct from a strict source/sink model (Fig. 3A).

The connectivity structure of H1N1 viruses follows a similar model of global circulation when the entire 14.25 years are analyzed together. More specifically, the Americas and Europe appear as a highly connected subnetwork (Figure S18B). Diversity waves originate from Asia in the northern hemisphere (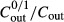
 = 1.00) and end up essentially in Oceania two quarters later (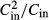
 = 1.00) with some spread to Europe, North America, and South America (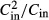
 = 0.25). At the same time, Oceania becomes the main distributor (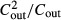
 = 0.75) and Asia turns into the main collector two quarters later (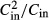
 = 1.00). The outgoing connectivity pattern in Fig. 2E matches perfectly the incoming pattern observed in Fig. 2H, hence closing the loop. Again, this pattern of connectivity of H1N1 diversity closely matches the one described previously, including the role of Asia (Russell et al. 2008)—a role that here is reduced to that of a point on a circuit. Yet, this average pattern is dominated by the presence of the H1N1/09 pandemic sequences. Indeed, without these sequences, Asia acts as the first distributor node with peak diversity in Q4 (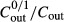
 = 1.00; Figure S13). Two quarters later, Oceania becomes the main collector (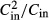
 = 1.00) followed in importance by North America (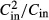
 = 0.33), while this latter region act as a distributor (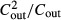
 = 1.00). Diversity waves then end up in Asia (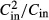
 = 1.00), where the wave started, while Europe and especially North America act as sinks. The outgoing connectivity pattern in Fig. 2I is almost a perfect negative of the incoming pattern observed in Fig. 2L, except for the role of Oceania. As a result, before the emergence of the H1N1/09 pandemic sequences, the spread of H1N1 diversity followed a strict source/sink model, emanating from Asia and ending up mostly outside of it (North America and Europe; no data available for South America), which is consistent with previous models (Rambaut et al. 2008; Russell et al. 2008) (both references included post-2005 data). The emergence of H1N1/09 and its sustained transmission since then may therefore be linked to a change in the global transmission network of H1N1, from a strict source/sink model to a global continuous circulation pattern just like H3N2. This shift in H1N1 global transmission network may be related to the series of reassortments that led up to the 2009 pandemic (Smith et al. 2009) and to chance reseeding. However, more data would be required to test this interplay between shifts of global circulation patterns and reassortment, or, alternatively, the hypothesis that global continuous circulation prevents the emergence of highly pathogenic strains by eliminating chance reseeding. Because all the results above rely on genetic diversity *ν*, the same analyses were also performed in terms of nucleotide diversity *π*; these additional analyses led to similar correlation patterns (Figures S19–S21) and global maps (Table S3 and Figure S22).

**Figure 4:**
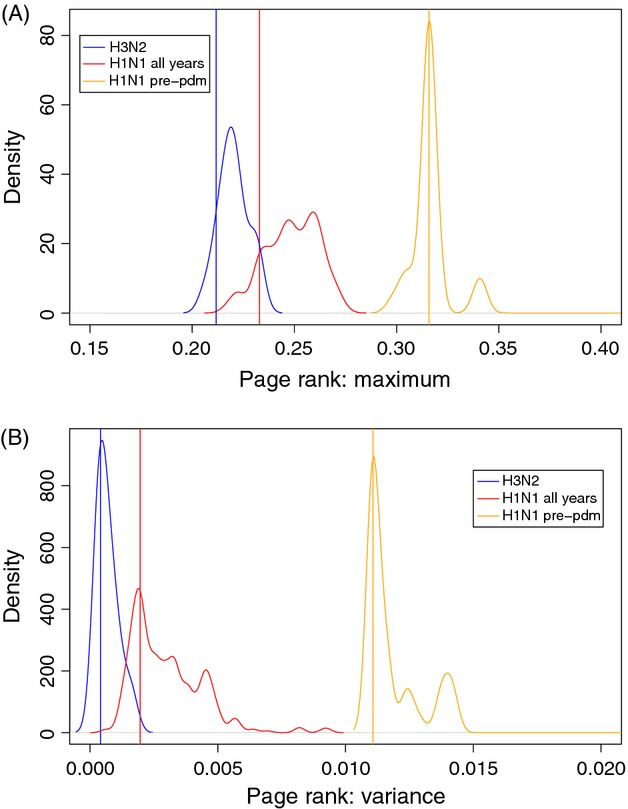
Comparison of connectivity networks based on the PageRank algorithm. (A) Sampling distribution of maximum PageRank eigenvalues for H3N2 (blue), H1N1 (all years; red) and H1N1 (prepandemic, up to Q4/2008; orange) over the five WHO regions. (B) Sampling distribution of the variance of PageRank eigenvalues for the same networks. These distributions were obtained by rewiring each network 10^5^ times. Vertical bars show observed values.

The significance of the difference between circulation patterns of H3N2 and prepandemic H1N1/09 diversities was assessed by computing the PageRank (PR) eigenvalues of the five WHO regions based on the reconstructed networks (Figure S18). If all the nodes had the same connectivity, their PR eigenvalues would all be equal and therefore would all have a variance of zero. Here, both the maximum PR eigenvalue (Fig. 4A) and the PR eigenvalue variance (Fig. 4B) have sampling distributions that overlap for H3N2 and H1N1 (all years), while the same distributions for prepandemic H1N1 do not, hereby showing that the shift in H1N1 connectivity network is highly significant (with 10^5^ replicates: *P* < 10^−5^).

This significant shift may be linked to the dominance and persistence of these subtypes through time. It would indeed make sense that if a given subtype circulates around the world through calendar years, using the same region both as a source and a sink, then this subtype is likely to persist over the years. Critically, this persistence would not be local (see Figure S2 in Russell et al. 2008), but nonlocal after continuous global circulation—and change through mutation and reassortment. Note that the term *circulation* is used here in its literal form, meaning ‘moving in a circular path’ (Stevenson and Lindberg 2010). In this case, no particular region acts exclusively as a source, which is the pattern also described by Bahl et al. (2011). If on the other hand a different subtype or strain goes through repeated emergence in one region and dies out at the end of the year in a different region (the source/sink model: Russell et al. 2008; Rambaut et al. 2008), then its persistence is more uncertain as reseeding is required every year with strains that were potentially not those circulating in the previous season. The results presented here are consistent with this intuition: H3N2 has been the dominant subtype in human populations since 1968 and has exhibited a continuous global circulation for at least the last 14 years; before 2009, H1N1 was not the dominant subtype and followed the source/sink model; since the emergence of the pandemic H1N1/09, this subtype is undergoing global continuous circulation and cocirculates with H3N2.

### Relationship between diversity and prevalence

In the context of these connectivity networks, one may wonder how the circulating waves of diversity around the world affect the health of human populations. Prevalence data are easily available for the USA, so that it is straightforward to test for the existence of a relationship between prevalence and genetic diversity. Let us assume that US prevalence data are representative of the whole of North America, an assumption that may not hold, but one that is likely to be valid here as most of the North American sequences are from the USA. From there, Figure S23 clearly demonstrates that prevalence is at its highest when diversity is at its lowest point (H1: *t* = −3.61, *P* = 0.0018, Figure S23A; H3: *t* = −3.44, *P* = 0.0016, Figure S23B). These results are consistent with previous reports of decreased genetic diversity after the early period of the season (Creanza et al. 2010). More fundamentally, these results suggest that an increase in prevalence is linked to the emergence of a dominant viral genotype, which is not at the forefront of the circulating diversity wave, but slightly behind (Lehe et al. 2012).

While the general approach described here supports previous results both in terms of global spread of influenza viruses and of prevalence cycles in relation to genetic diversity, one important caveat should be highlighted. Because the results are based on seasonality components extracted from time series over a certain period of time, inferences are sensitive to the time period considered, and to potential ‘irregularities’ such as the 2009 pandemic H1 viruses. In ‘regular’ years, genetic diversity peaks during the winter months in the northern hemisphere (Rambaut et al. 2008). When the data are analyzed up to Q4/2008, the results are consistent with this previous inference for both H1 (Figure S24A) and H3 (Figure S24B) viruses. However, the inclusion of data collected since 2009 biases the time series analysis, shifting the diversity peak for H3 (Figure S24C) and H1 by one quarter. This example reinforces the point made previously (Bahl et al. 2011) that trends averaged over long periods of time do not necessarily predict particular events accurately and that the evolution of influenza viruses still exhibits a fair amount of contingency (Nelson et al. 2006).

**Figure 5:**
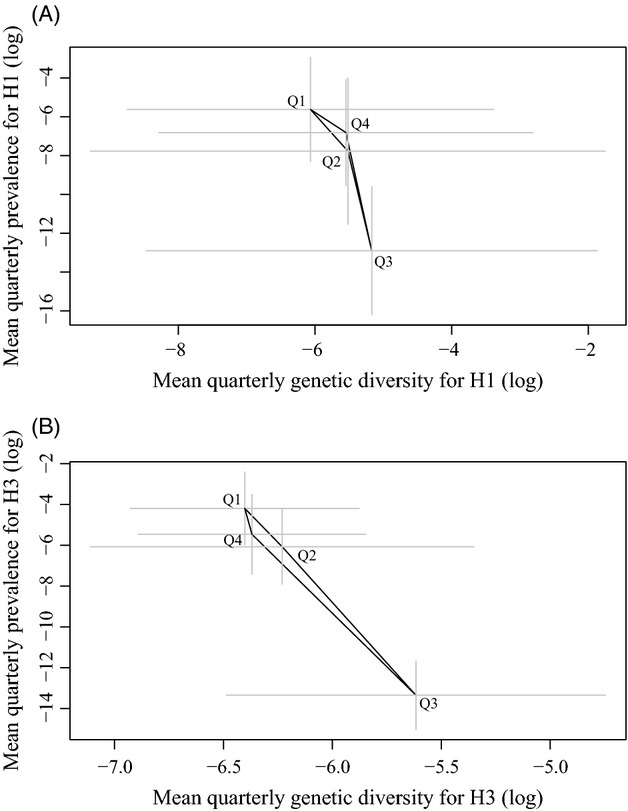
Prevalence–diversity cycles of influenza viruses through a full year. Quarterly mean prevalence and mean genetic diversity on a log_10_-log_10_ scale (A) for H1 viruses, (B) for H3 viruses. Each quarter is indicated with the letter Q (Q1 to Q4). Gray bars represent one standard deviation of the mean.

To circumvent this issue and represent which quarter is associated with low diversity and high prevalence, the quarterly means were computed for both diversity and prevalence; in this way, the results are independent of the averaged seasonality patterns extracted from the time series analysis. Figure 5 shows that for both subtypes H1 and H3 prevalence peaks in Q1 when genetic diversity is at its lowest, while Q3 represents the lowest prevalence quarter but has the highest diversity. Based on this figure, it could be suggested that H1 viruses cycle counterclockwise in a prevalence/diversity space (Fig. 5A), while H3 viruses cycle the other way around (Fig. 5B). However, error bars are so wide that making such an inference would demand surveying more years.

This latter result has a further implication: If increases in prevalence can be traced back to the emergence of a viral genotype that will become dominant later in the season, and that viral diversity circulates around the globe, then similar waves of prevalence around the world should also be expected. Data from the WHO (http://www.who.int/influenza/surveillance_monitoring/updates) show that different regions have peak prevalence at different times of the year, but a more systematic study should be undertaken. An option would be to use FluNet, available at http://apps.who.int/globalatlas/dataQuery/default.asp. However, prevalence cannot be computed as the total number of individuals tested relates to the week when processed, while the number of positives relates to when the specimens were collected (WHO GISRS Team, pers. comm. by email; May 22, 2012), an issue probably encountered by others (Finkelman et al. 2007) who instead worked with incidence (the number of positives divided by the census size of the population studied).

## Conclusions

While numerous models have been developed to reconstruct the spread of viruses, either in a parsimonious (Wallace et al. 2007) or in a sound statistical framework (Lemey et al. 2009), their use can be computationally demanding when analyzing thousands of sequences. Instead of reconstructing a single phylogenetic tree with these models, I analyzed a total of 15 335 sequences spread over 14.25 years of data stratified spatially (WHO regions) and temporally (quarters) to assess the relationship existing between the seasonal components of viral diversity for two influenza subtypes, H3N2 and H1N1. The key point here is that this long-term analysis was performed in the absence of a *complete* reconstruction of viral ancestry over the whole period. While this novel approach does not negate a full phylogenetic analysis, the results presented here, based on simple estimates of diversity, are amendable to analyze very large data sets and are globally consistent with previous studies, which were based on complete phylogenetic analyses (Rambaut et al. 2008; Russell et al. 2008) but much smaller data sets.

One of the strengths of the method presented here is that it relies on a stratified design, which by construction does not assume spatial or time homogeneity of mutation rates (only local homogeneity, within a slice of time and space, is required). Models incorporating episodic shifts of mutation or selection rates have recently been developed (Murrell et al. 2012a, b2012b), but may prove less robust than the model-free (not likelihood-based) approach described here. Although the HA gene is under strong pressure to evolve adaptively, only the information at the DNA level was considered here; a more sophisticated summary or set of summary statistics might be used, such as nonsynonymous to synonymous rate ratios (Goldman and Yang 1994; Muse and Gaut 1994). However, this would shift the focus from uncovering the spread of genetic or nucleotide diversity to estimating episodic changes in selective pressures through (geographic) space. The approach also has a number of limitations. Among them: (i) the lack of confidence intervals for relative connectivities, in particular when the number of nodes (regions) is small, or (ii) the dependence on the data used. In this latter situation, wavelet analysis (Tom et al. 2012; Cheng et al. 2013) or short-time Fourier transforms might prove more robust to ‘nonregular’ seasons such as 2009–2010 for H1 viruses. Because the analysis relies on a particular summary statistic such as genetic diversity (*ν*), it can be expected that the results are sensitive to this particular choice; however, I showed that using nucleotide diversity (*π*) gives a very similar map at the outcome of the network analysis, hereby suggesting that the algorithm is robust to the actual choice of summary statistic. Further work would be required to validate this preliminary result, based on only two summary statistics.

Although the global transmission dynamics of influenza viruses in humans are driven by a combination of seasonal stimuli and mechanisms (Tamerius et al. 2011), the results presented here show that long-term trends exist. I showed in particular that diversity of dominant subtypes, that is, those that show a sustained global transmission network over several seasons, circulate continuously around the world, even if some regions such as South America appear to act as sinks (Fig. 3B). On the other hand, the diversity of prepandemic H1N1 viruses showed a global transmission network consistent with a strict source/sink model, without continuous circulation (Fig. 3A). This absence of continuous circulation may explain why H1N1 was not the dominant subtype before the emergence of the pandemic H1N1/09. Irrespective of shifts in global transmission networks, the data presented here also show that prevalence is negatively correlated with diversity, at least in the USA. This result suggests that (i) epidemics are caused by a dominant viral strain and that (ii) monitoring genetic or nucleotide diversity in real time could be used as a proxy for determining prevalence of influenza A viruses circulating in human populations.

## Data archiving

Data used in this study were obtained from the IVR. Alignments and estimated trees are available in the Online Supplementary Materials.

## Supporting Information

**Figure S1.** Geographic distribution of the sequences collected for this study. **Figure S2.** Distribution of the data analyzed. **Figure S3.** Number *N* of HA sequences analyzed. **Figure S4.** Prevalence of influenza infections by subtype in the USA. **Figure S5.** Distribution of bootstrap values over all the data sets analyzed. **Figure S6.** Correlation between estimates of nucleotide diversity π and of genetic diversity ν. **Figure S7.** Genetic diversity (ν) for H1 and H3 viruses across the five regions. **Figure S8.** Time series of genetic diversity ν for H1 and H3 viruses from Asia. **Figure S9.** Time series of genetic diversity ν for H1 and H3 viruses from Europe. **Figure S10.** Time series of genetic diversity ν for H1 and H3 viruses from North America. **Figure S11.** Time series of genetic diversity ν for H1 and H3 viruses from Oceania. **Figure S12.** Time series of genetic diversity ν for H1 and H3 viruses from South America. **Figure S13.** Seasonality of genetic diversity ν for H1 and H3 viruses across the five regions. **Figure S14.** Time series of genetic diversity ν for H1 and H3 viruses. **Figure S15.** Autocorrelation functions for genetic diversity ν in prepandemic H1 viruses (up to Q4/2008) across the five regions studied here. **Figure S16.** Autocorrelation functions for genetic diversity ν in H1 viruses across the five regions studied. **Figure S17.** Autocorrelation functions for genetic diversity ν in H3 viruses across the five regions studied. **Figure S18.** Global absolute connectivity of influenza genetic diversity ν. **Figure S19.** Autocorrelation functions for nucleotide diversity π in prepandemic H1 viruses (up to Q4/2008) across the five regions studied here. **Figure S20.** Autocorrelation functions for nucleotide diversity π in H1 viruses across the five regions studied. **Figure S21.** Autocorrelation functions for nucleotide diversity π in H3 viruses across the five regions studied. **Figure S22.** Global relative connectivity of influenza nucleotide diversity π through time. **Figure S23.** Prevalence as a function of genetic diversity in the USA. **Figure S24.** Seasonality of genetic diversity and of prevalence in the USA.

**Table S1.** Correlation of the timing of m diversity peaks between regions by subtypes. **Table S2.** Connectivity analysis of the five regions in terms of genetic diversity ν. **Table S3.** Connectivity analysis of the five regions in terms of nucleotide diversity π.
