# Supplementary material for: Inferring influenza global transmission networks without complete phylogenetic information
Source: Evol Appl. 2014 Jan 2;7(3):403–12. doi: 10.1111/eva.12138 (PMC3962300; doi:10.1111/eva.12138)
Supplement: Supplementary file 1 — Figure S1. Geographic distribution of the sequences collected for this study. Figure S2. Distribution of the data analyzed. Figure S3. Number N of HA sequences analyzed. Figure S4. Prevalence of influenza infections by subtype in the USA. Figure S5. Distribution of bootstrap values over all the data sets analyzed. Figure S6. Correlation between estimates of nucleotide diversity π and of genetic diversity ν. Figure S7. Genetic diversity (ν) for H1 and H3 viruses across the five regions. Figure S8. Time series of genetic diversity ν for H1 and H3 viruses from Asia. Figure S9. Time series of genetic diversity ν for H1 and H3 viruses from Europe. Figure S10. Time series of genetic diversity ν for H1 and H3 viruses from North America. Figure S11. Time series of genetic diversity ν for H1 and H3 viruses from Oceania. Figure S12. Time series of genetic diversity ν for H1 and H3 viruses from South America. Figure S13. Seasonality of genetic diversity ν for H1 and H3 viruses across the five regions. Figure S14. Time series of genetic diversity ν for H1 and H3 viruses. Figure S15. Autocorrelation functions for genetic diversity ν in prepandemic H1 viruses (up to Q4/2008) across the five regions studied here. Figure S16. Autocorrelation functions for genetic diversity ν in H1 viruses across the five regions studied. Figure S17. Autocorrelation functions for genetic diversity ν in H3 viruses across the five regions studied. Figure S18. Global absolute connectivity of influenza genetic diversity ν. Figure S19. Autocorrelation functions for nucleotide diversity π in prepandemic H1 viruses (up to Q4/2008) across the five regions studied here. Figure S20. Autocorrelation functions for nucleotide diversity π in H1 viruses across the five regions studied. Figure S21. Autocorrelation functions for nucleotide diversity π in H3 viruses across the five regions studied. Figure S22. Global relative connectivity of influenza nucleotide diversity π through time. Figure S23. Prevalence as a f [file eva0007-0403-sd1.pdf]

Supplementary Text

Data pre-processing

After retrieving the sequence data, an in-house Perl script was used to split these sequences by quarter (quarters running from January 1 to March 31 [week 1-13], April 1 to June 30 [week 14-26], July 1 to September 30 [week 27-39] and October 1 to December 31 [week 40-52]).

The original data set contained 9,694 H1 and 8,981 H3 sequences. After discarding sequences without information about collection month, 8,748 H1 and 6,587 H3 sequences were left in the analysis. Human sequences coming from inter-species transmission were not filtered out, as these typically divergent sequences carry some information and can be of critical epidemiological importance.

Sequence alignments

All the data sets were aligned with `Muscle` (Edgar, 2004) with default parameters. Sequences were not trimmed in order to conserve as many variable sites as possible upstream and downstream of the coding sequence. Because of the potential presence of noncoding sequences in the data and of out-of-frame data, alignments were performed directly on DNA instead of protein sequences (*e.g.*, Aris-Brosou, 2010; Abdussamad and Aris-Brosou, 2011). This was not problematic here as sequences within a quarter and a given region showed high levels of similarity. Alignments were visually inspected, with `JalView` (Waterhouse et al., 2009), misaligned sequences were removed (H1: HM625636, CY083655; H3: FJ769860, EU835537, EU642547, EU642548) and gaps were adjusted manually. The alignments used in this study are available from the journal's website.

Time series and network analyses

The phylogenetic analyses lead to the estimation of genetic diversity ν by quarter, subtype and WHO region. Within each subtype and each WHO region, these ν estimates can be conceived as "temporal" or time series running from 1996 to 2011. Each such time series was then decomposed into rend (m_t), the seasonal effect (s_t) and an error term (ϵ_t) as per equation (1) in the main text.

After this time series decomposition, the seasonality component of these time series was plotted in two equivalent ways: (i) as a times series plot and (ii) as correlograms derived from computation of autocorrelation functions (ACFs) among the WHO regions – see figures S16-S17. These autocorrelations were then used to determine significant time lags between all pairs of WHO regions. In the ACF plots, correlation coefficients are approximately normally distributed with a mean of $-\frac{1}{n}$ and a variance of $\frac{1}{n}$ for n observations (*i.e.*, for n quarters). Horizontal dotted lines on correlograms drawn at $-\frac{1}{n} \pm \frac{3}{\sqrt{n}}$ represent approximate significance at the 1% level when testing the null hypothesis that the correlation is 0 at a given lag (the default setting, at the 5% level, may be too liberal). With 14.25 years, we have 57 quarters so that the 1% bounds are at 0.38 and -0.42. A connection between two regions was then graphically inferred when the autocorrelation was significant for lags ≤ 2 quarters (a half year) as indicated in figures S16-S17. When > 1 correlation peaks existed, only the most significant peak was considered. This whole approach is akin to binarizing network connectivities, rather than using a weight connectivity matrix.

Because the ACFs are signed, it is possible to determine the direction of the lag, with for instance region R_2 peaking one quarter *after* region R_1. This would support the existence of a signed connection or *lag vector* that goes from R_1 to R_2 for a lag of one quarter. These lag vectors can then be plotted on a map (figure S18), from which

it becomes straightforward to compute the connectivities presented in table S2. These connectivities are then tallied as total connectivities for the total number of lag vectors at each WHO region (C_t in table S2). In turn, total connectivities can be decomposed into the number of lag vectors arriving at each WHO region (C_{in}) or departing from the same region (C_{out}). Note of course that $C_t = C_{in} + C_{out}$ for each region within each subtype. Relative connectivities are computed for each lag as described in the main text.

References

Abdussamad, J., and S. Aris-Brosou. 2011. The nonadaptive nature of the H1N1 2009 Swine Flu pandemic contrasts with the adaptive facilitation of transmission to a new host. BMC Evol Biol **11**:6.

Aris-Brosou, S. 2010. A simple measure of the dynamics of segmented genomes: An application to influenza. Lecture Notes in Computer Science **6398 LNBI**:149–160.

Edgar, R. C. 2004. MUSCLE: multiple sequence alignment with high accuracy and high throughput. Nucleic Acids Res **32**:1792–7.

Waterhouse, A. M., J. B. Procter, D. M. A. Martin, M. Clamp, and G. J. Barton. 2009. Jalview Version 2: a multiple sequence alignment editor and analysis workbench. Bioinformatics **25**:1189–91.

Supplementary Figures

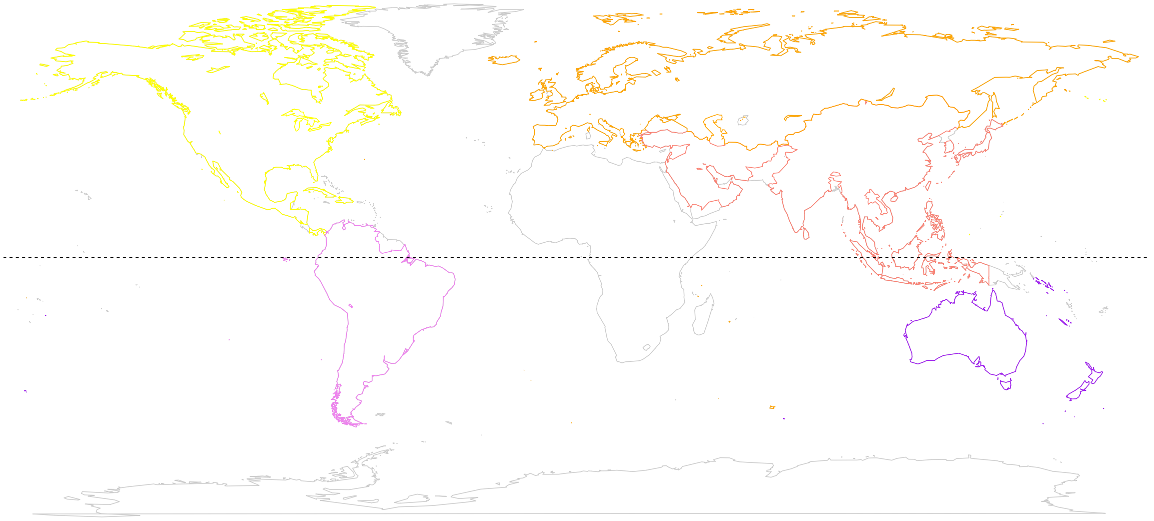

Figure S1. Geographic distribution of the sequences collected for this study. WHO regions are color-coded as: Asia (salmon), Europe (orange), North America (yellow), Oceania (purple) and South America (violet). Parts of the world in light gray have no or not enough data and were not considered here. The dashed horizontal line is the equator, the dotted lines correspond to the tropics (Cancer: 23.4378° N; Capricorn: 23.4378° S).

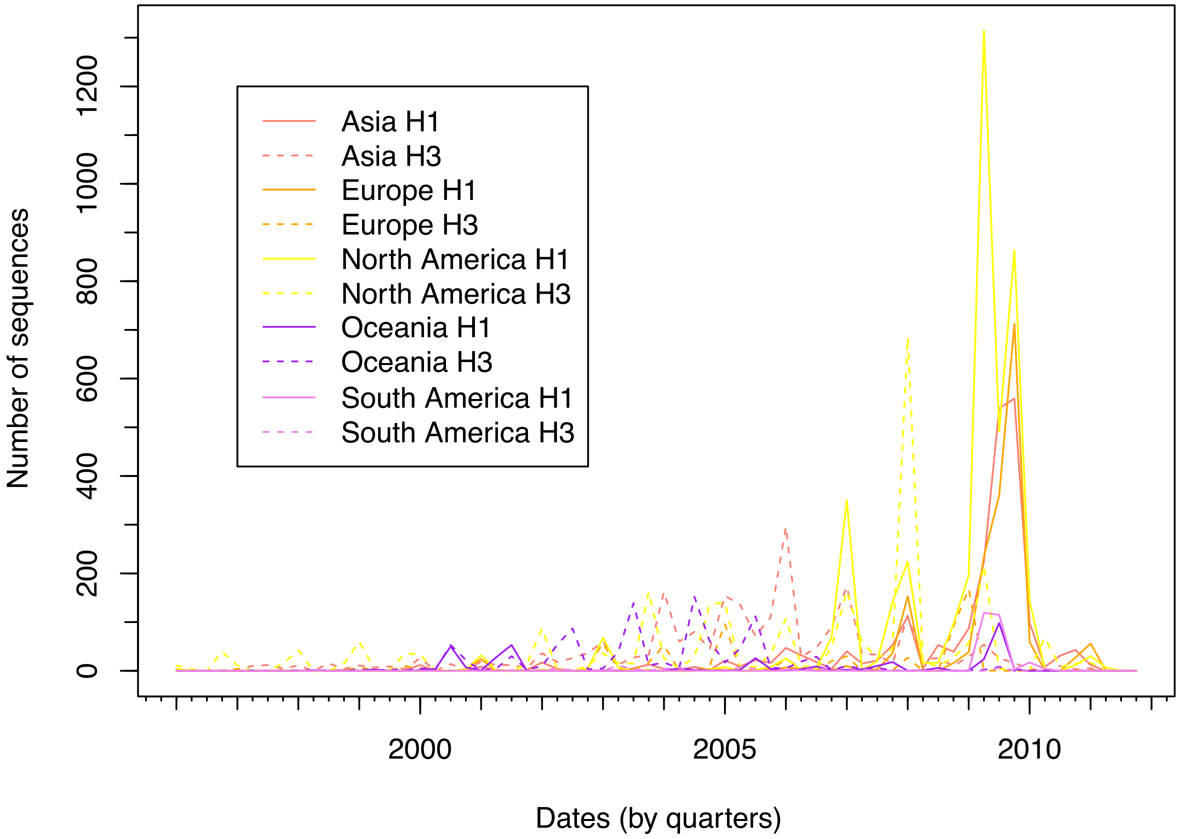

Figure S2. Distribution of the data analyzed. WHO regions are color-coded as in the inset. Solid lines represent H1 viruses, and broken lines represent H3 viruses.

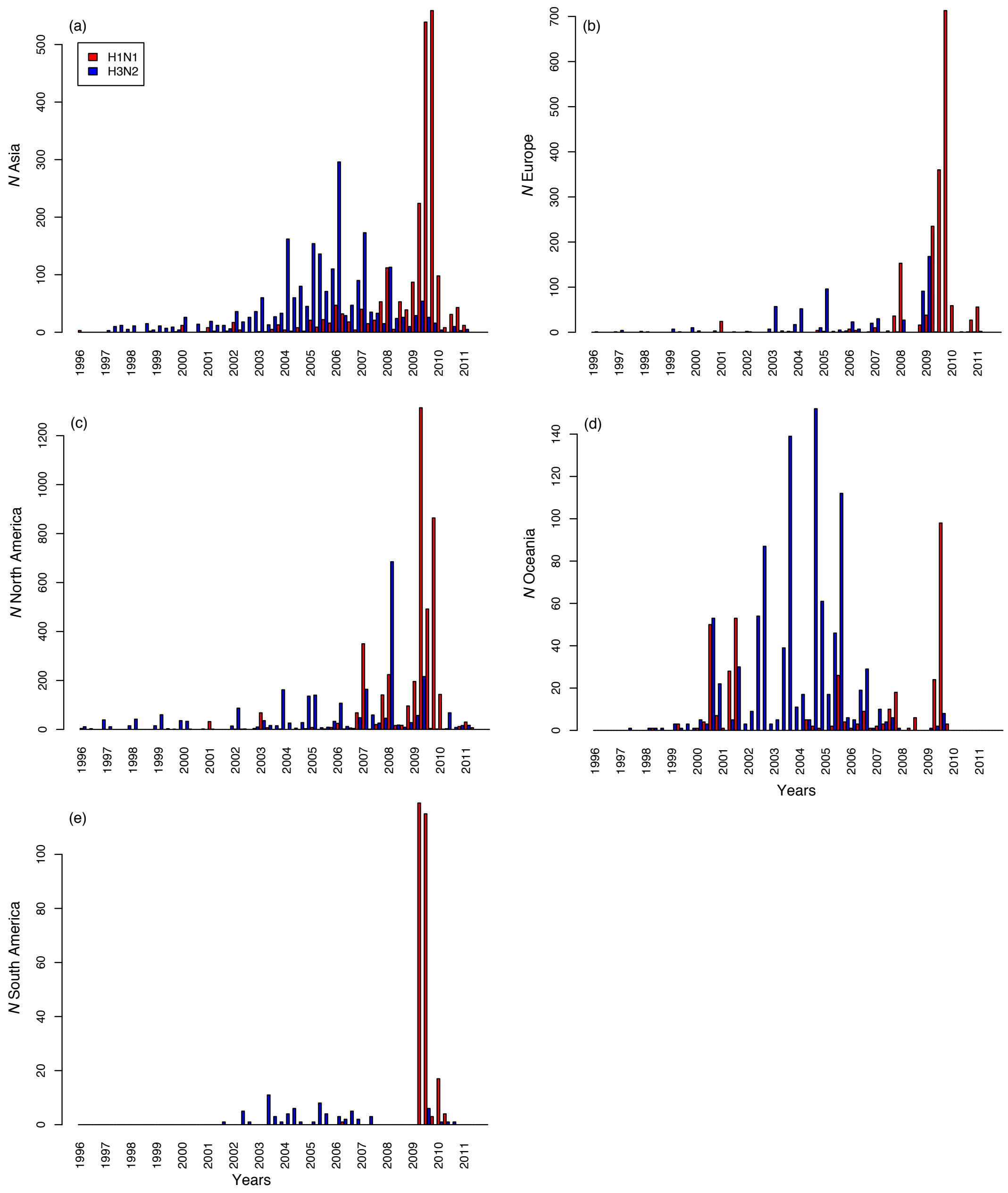

Figure S3. Number N of HA sequences analyzed. The five WHO regions analyzed here are shown as: (a): Asia; (b): Europe; (c): North America; (d): Oceania and (e): South America. Red bars represent H1 viruses, and blue bars represent H3 viruses.

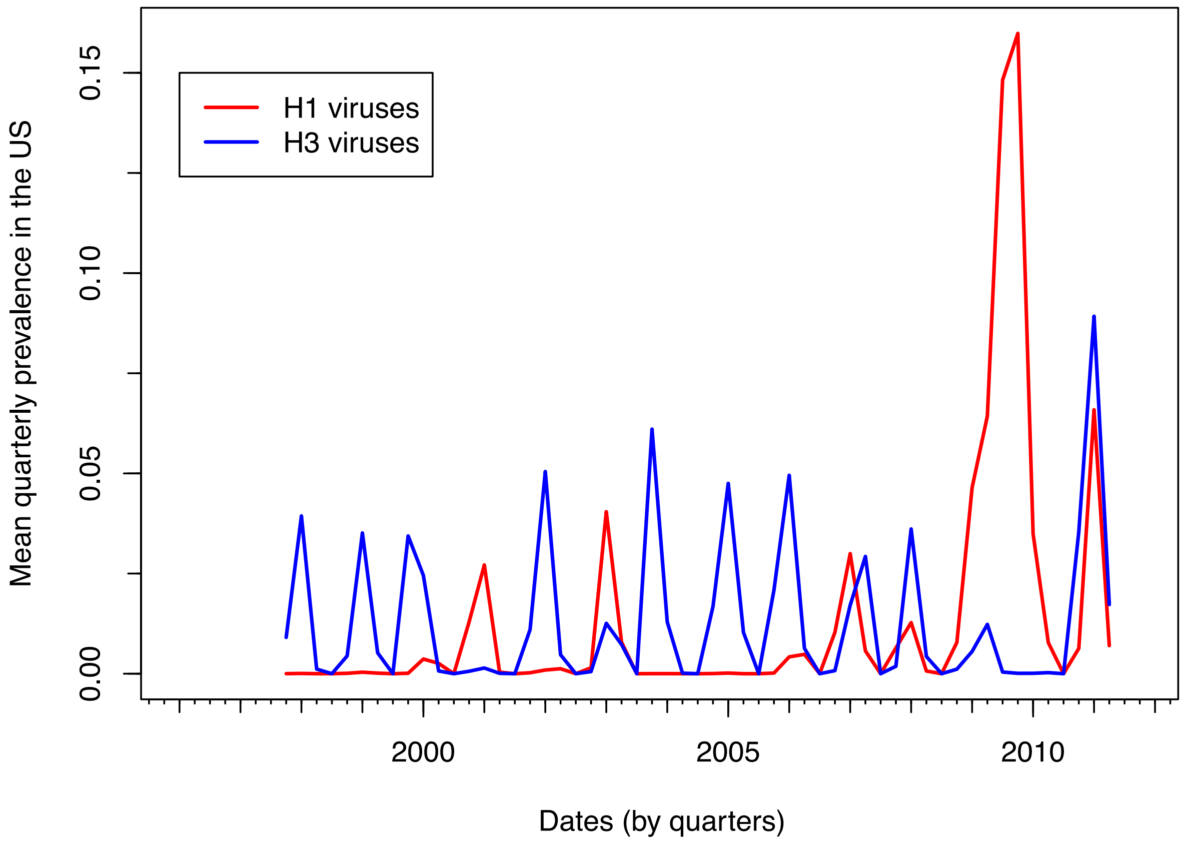

Figure S4. Prevalence of influenza infections by subtype in the US. Data for H1 viruses are in red, and in blue for H3 viruses. Data from the CDC.

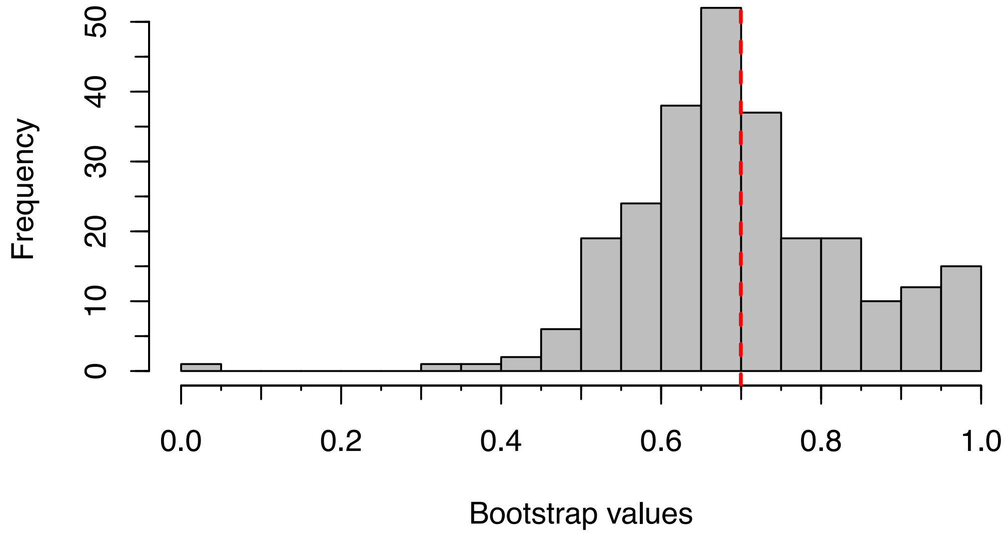

Figure S5. Distribution of bootstrap values over all the data sets analyzed. One hundred replicates were performed for each data set. The mean is showed by a vertical dotted red line ($\bar{B}_p = 0.6996$).

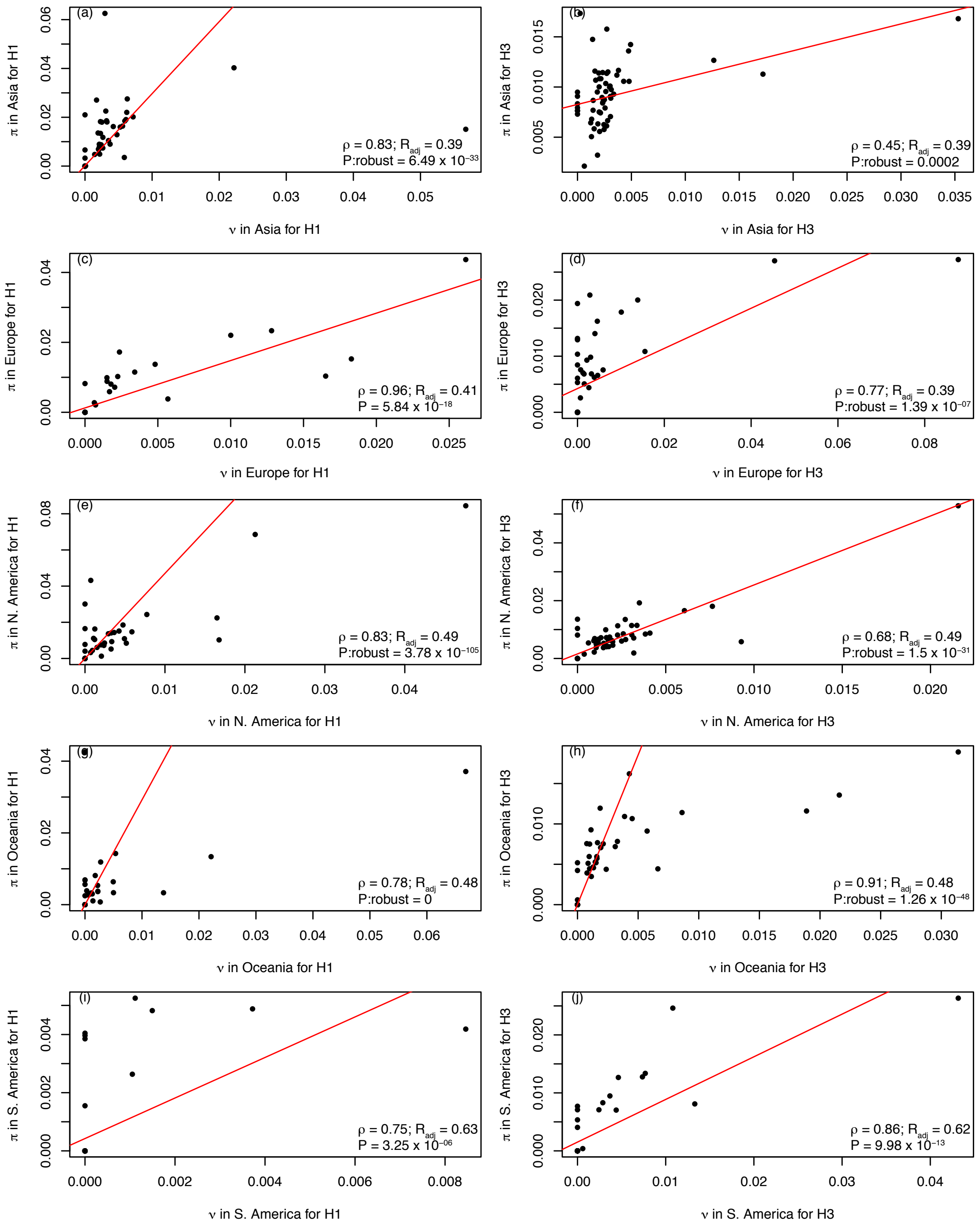

Figure S6. Correlation between estimates of nucleotide diversity π and of genetic diversity ν. Results are shown by region and subtypes: in Asia for (a) H1N1 and (b) H3N2, in Europe for (c) H1N1 and (d) H3N2, in North America for (e) H1N1 and (f) H3N2, in Oceania for (g) H1N1 and (h) H3N2 and in South America for (i) H1N1 and (j) H3N2. Robust linear regression were fitted to the data (red lines). Correlation coefficients (Spearman's rank ρ), adjusted R-squared (R_{adj}) and P-values are shown.

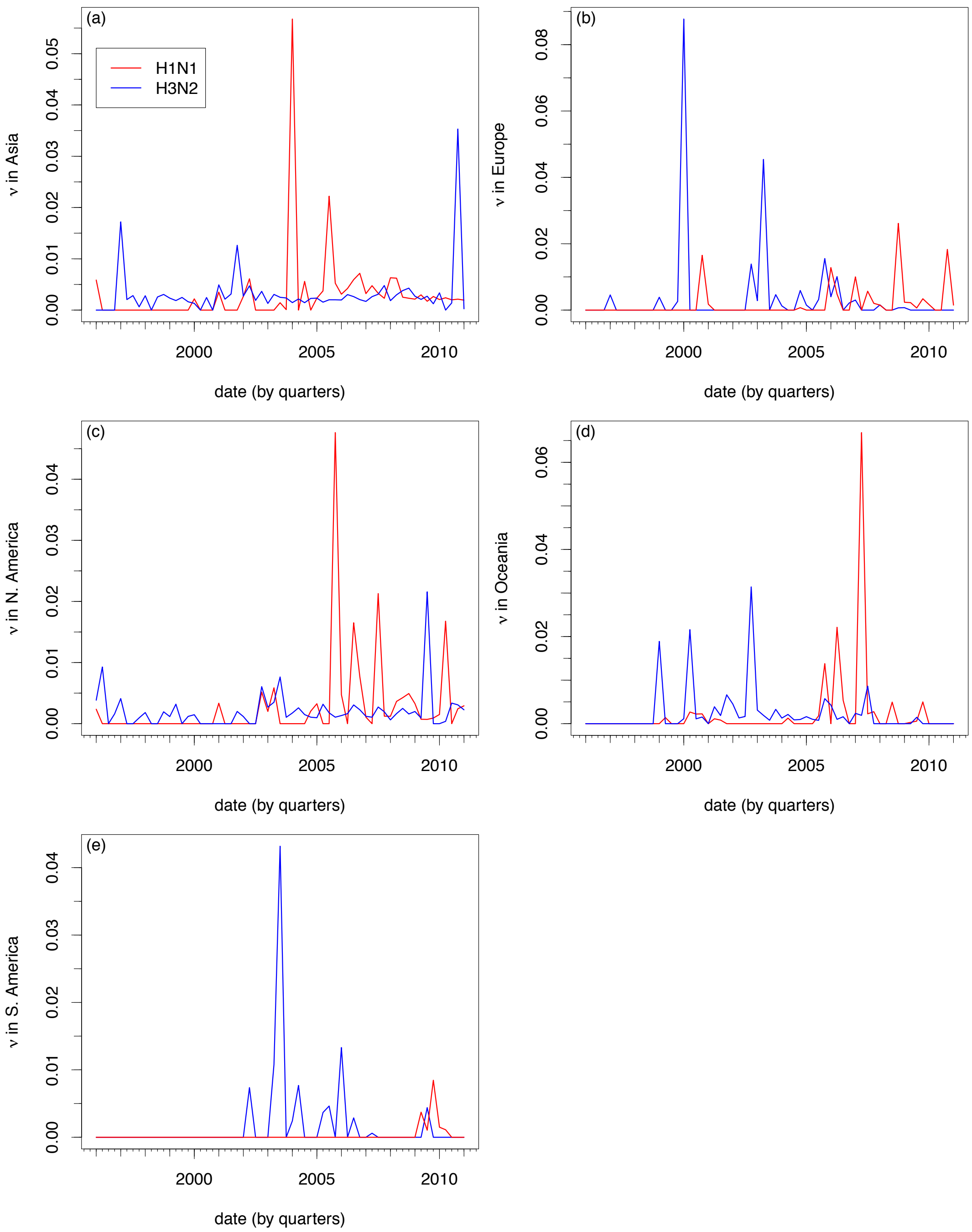

Figure S7. Genetic diversity (ν) for H1 and H3 viruses across the five regions. The five regions analyzed here are shown as: (a): Asia; (b): Europe; (c): North America; (d): Oceania and (e): South America. Data for H1 viruses are in red, and in blue for H3 viruses.

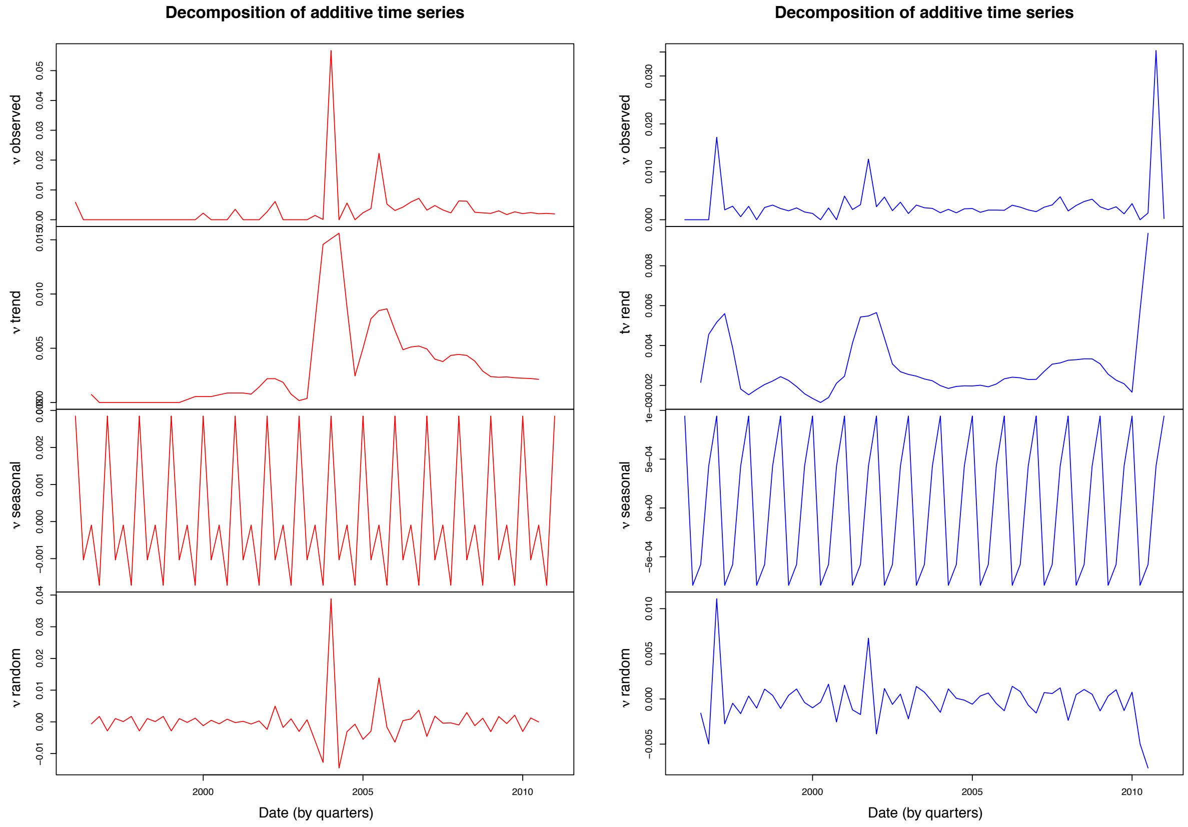

Figure S8. Time series of genetic diversity ν for H1 and H3 viruses from Asia. Red lines represent H1 viruses (left), and blue lines represent H3 viruses (right). See Methods for details.

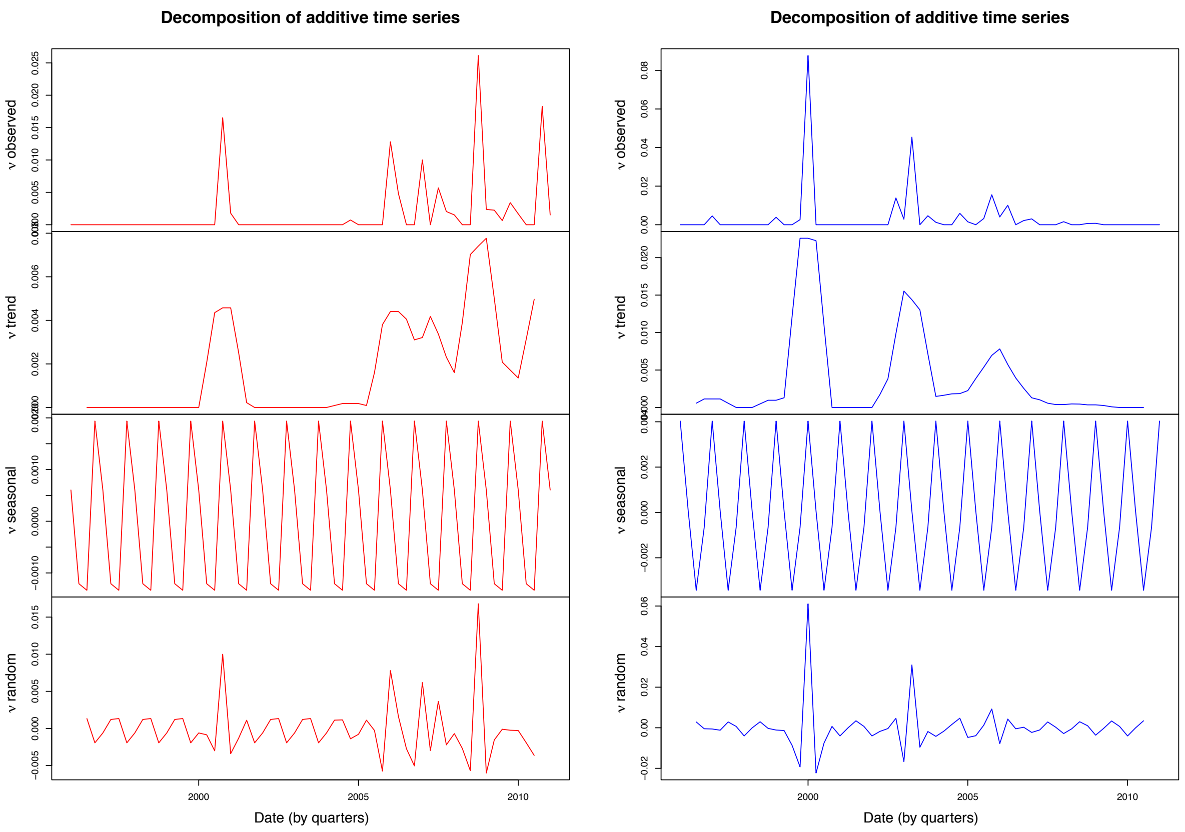

Figure S9. Time series of genetic diversity ν for H1 and H3 viruses from Europe. Red lines represent H1 viruses (left), and blue lines represent H3 viruses (right). See Methods for details.

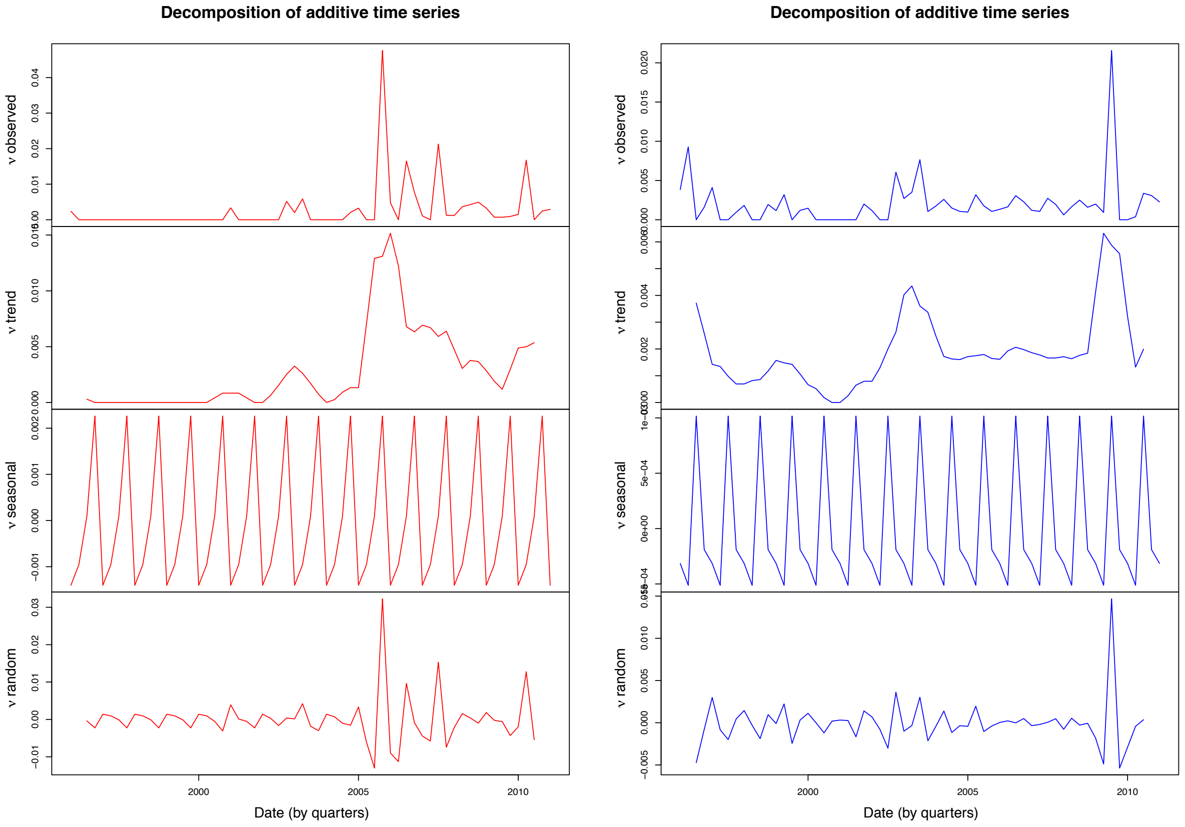

Figure S10. Time series of genetic diversity ν for H1 and H3 viruses from North America. Red lines represent H1 viruses (left), and blue lines represent H3 viruses (right). See Methods for details.

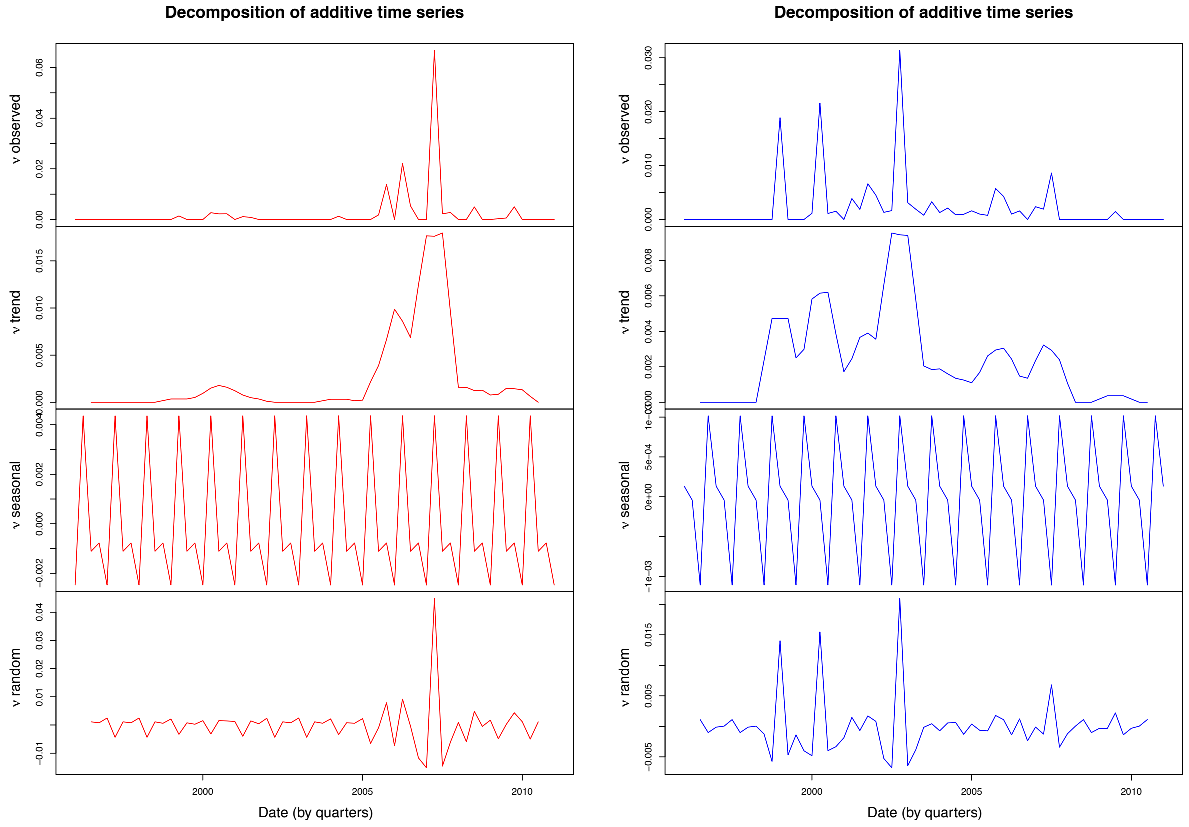

Figure S11. Time series of genetic diversity ν for H1 and H3 viruses from Oceania. Red lines represent H1 viruses (left), and blue lines represent H3 viruses (right). See Methods for details.

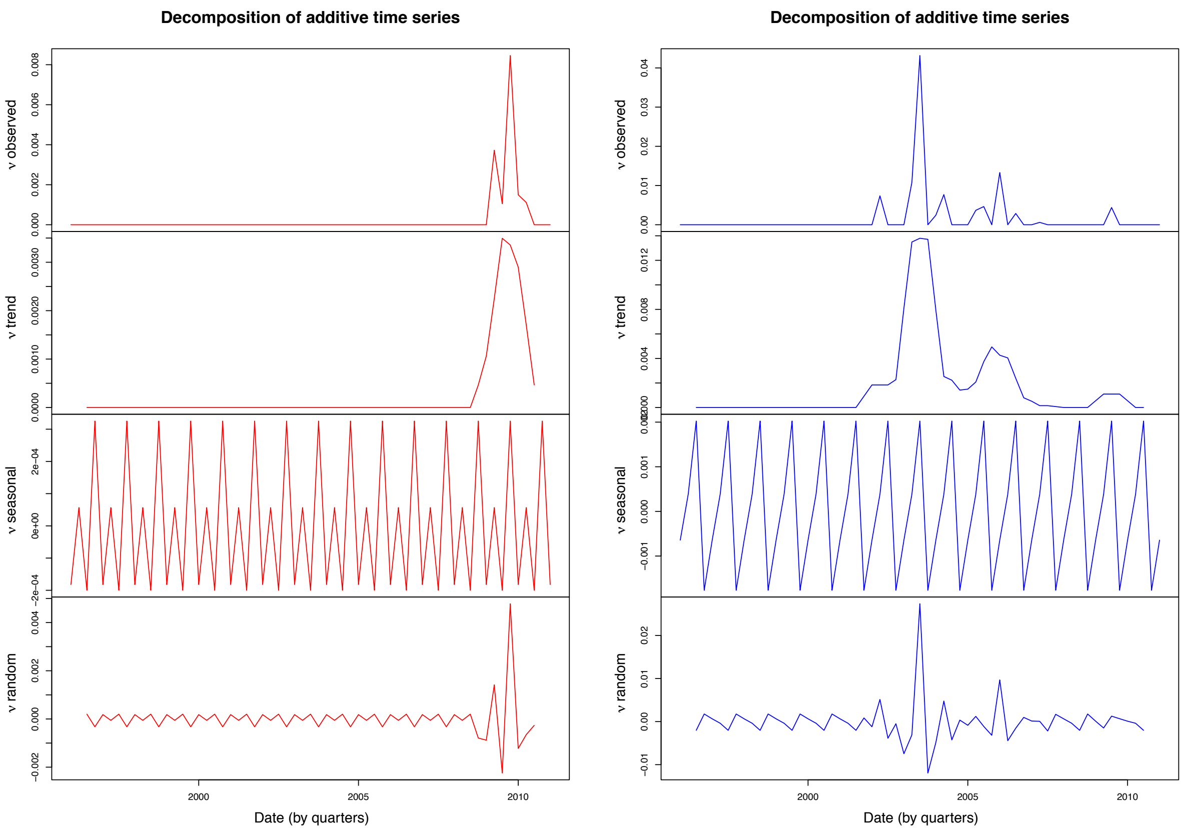

Figure S12. Time series of genetic diversity ν for H1 and H3 viruses from South America. Red lines represent H1 viruses (left), and blue lines represent H3 viruses (right). See Methods for details.

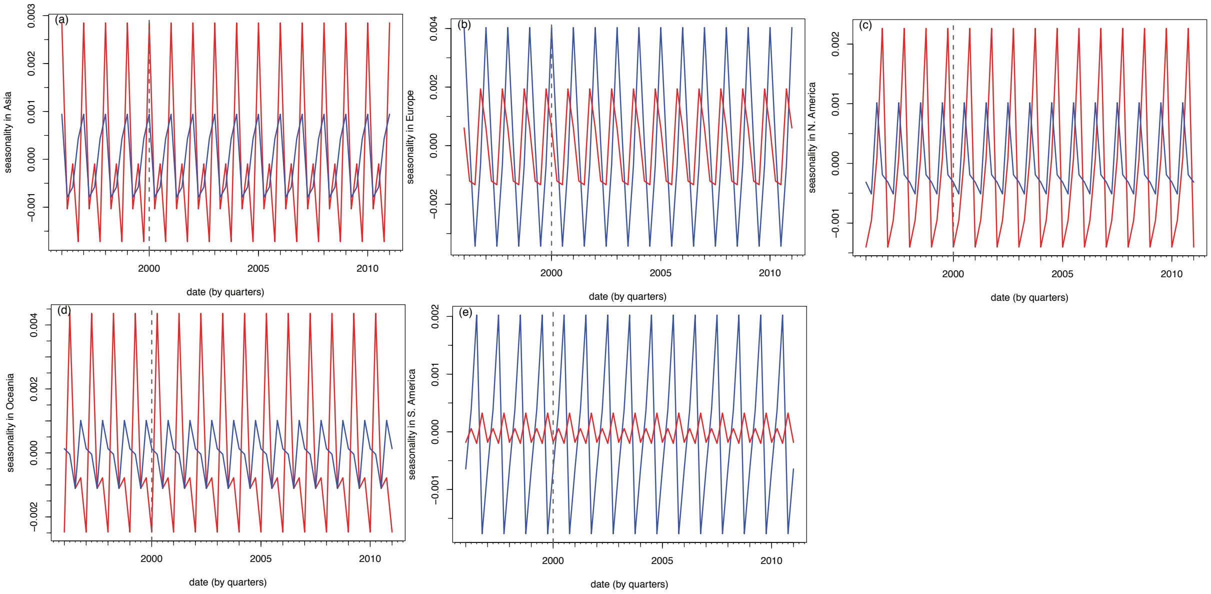

Figure S13. Seasonality of genetic diversity ν for H1 and H3 viruses across the five regions. The five regions analyzed here are shown as: (a): Asia; (b): Europe; (c): North America; (d): Oceania and (e): South America. Data for H1 viruses are in red, and in blue for H3 viruses. A vertical line is drawn arbitrarily at the beginning of Q1/2000 for reference.

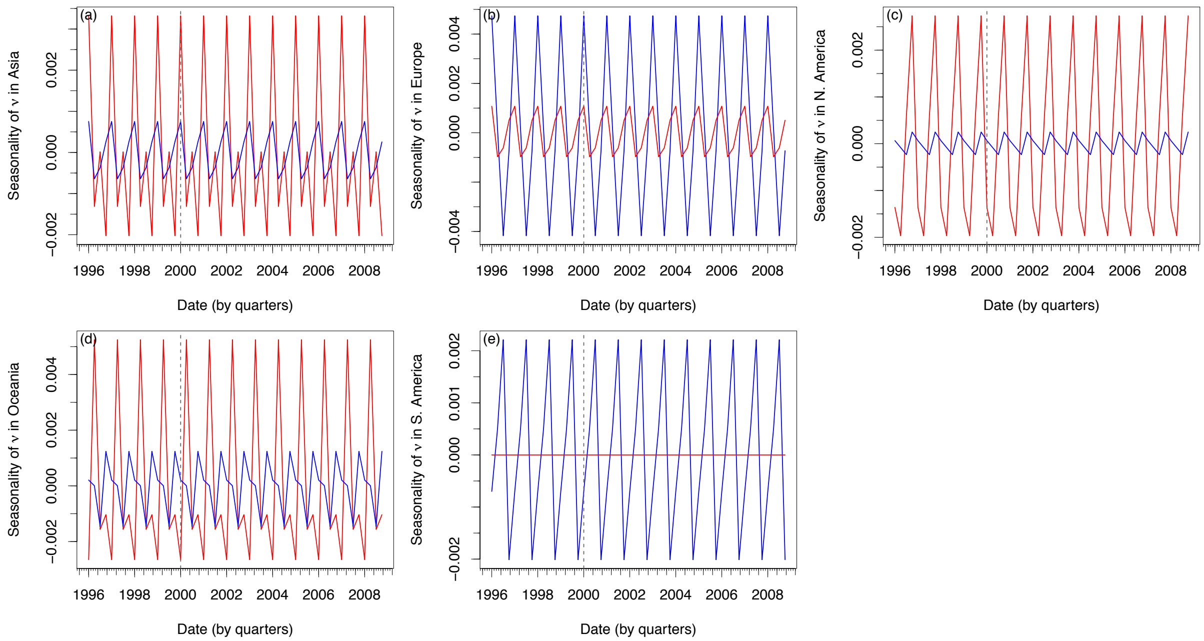

Figure S14. Time series of genetic diversity ν for H1 and H3 viruses. Only pre-pandemic data are analyzed (up to Q4/2008). The five regions analyzed here are shown as: (a): Asia; (b): Europe; (c): North America; (d): Oceania and (e): South America. Data for H1 viruses are in red, and in blue for H3 viruses.

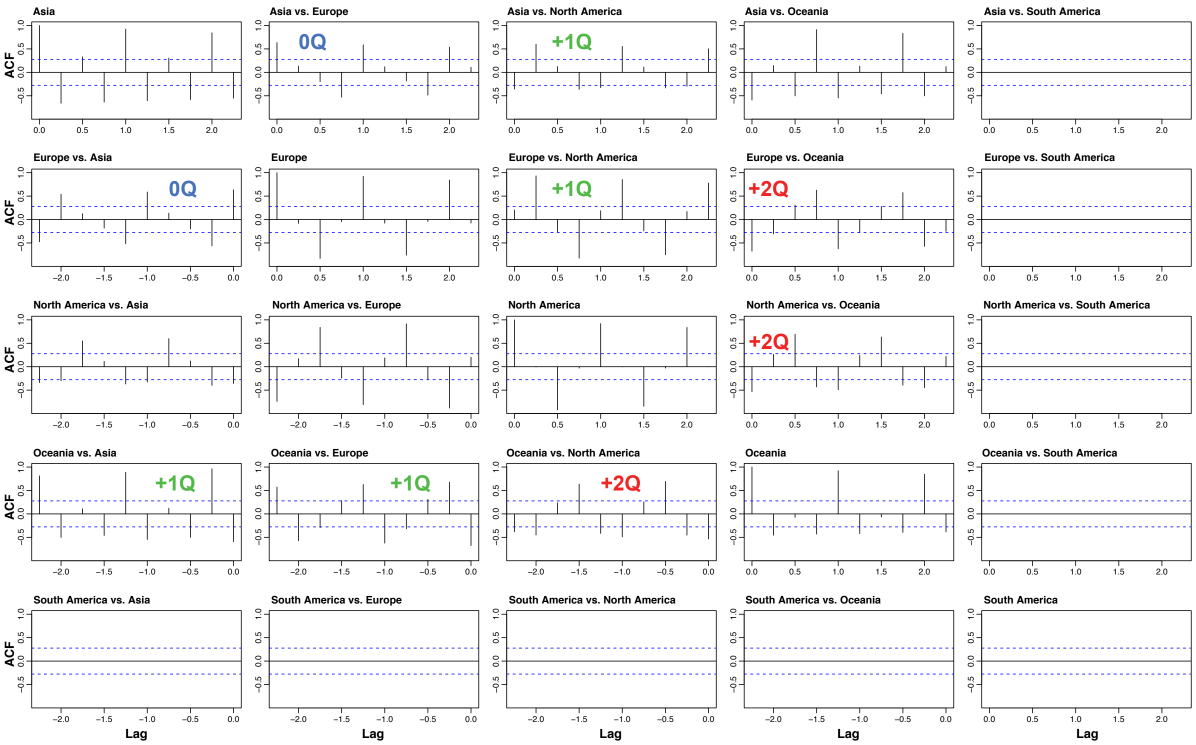

Figure S15. Autocorrelation functions for genetic diversity ν in pre-pandemic H1 viruses (up to Q4/2008) across the five regions studied here. Regions are ordered as follows: Asia, Europe, North America, Oceania and South America. The time unit for lag is one year. Significant lags are color coded: 0: blue; 1: green; 2: red. Horizontal broken lines: 99% significance level.

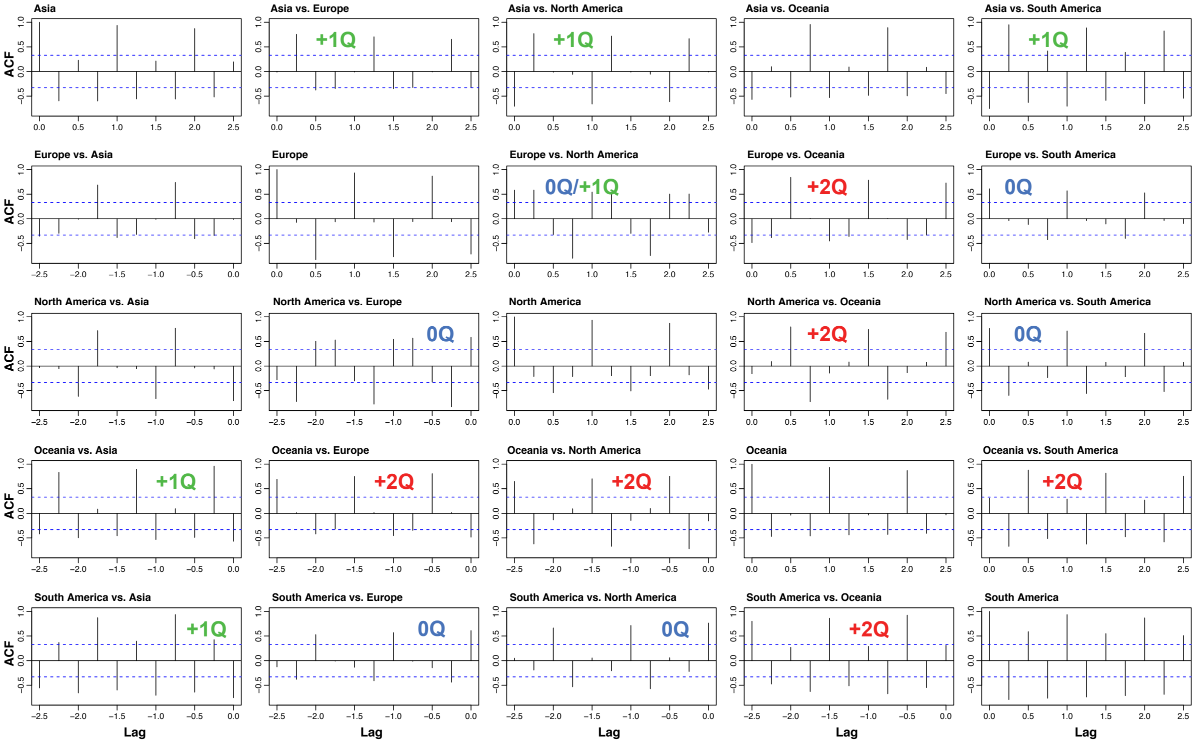

Figure S16. Autocorrelation functions for genetic diversity ν in H1 viruses across the five regions studied. Regions are ordered as follows: Asia, Europe, North America, Oceania and South America. The time unit for lag is one year. Significant lags are color coded: 0: blue; 1: green; 2: red. Horizontal broken lines: 99% significance level.

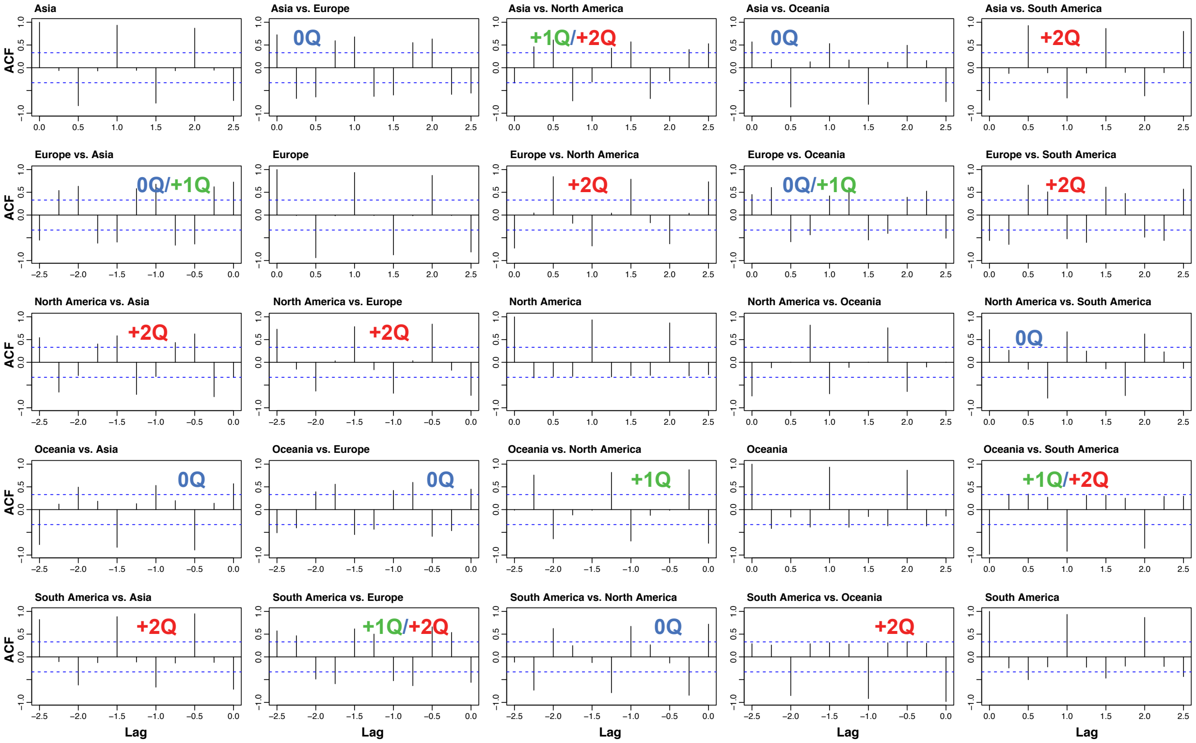

Figure S17. Autocorrelation functions for genetic diversity ν in H3 viruses across the five regions studied. Regions are ordered as follows: Asia, Europe, North America, Oceania and South America. The time unit for lag is one year. Significant lags are color coded: 0: blue; 1: green; 2: red. Horizontal broken lines: 99% significance level.

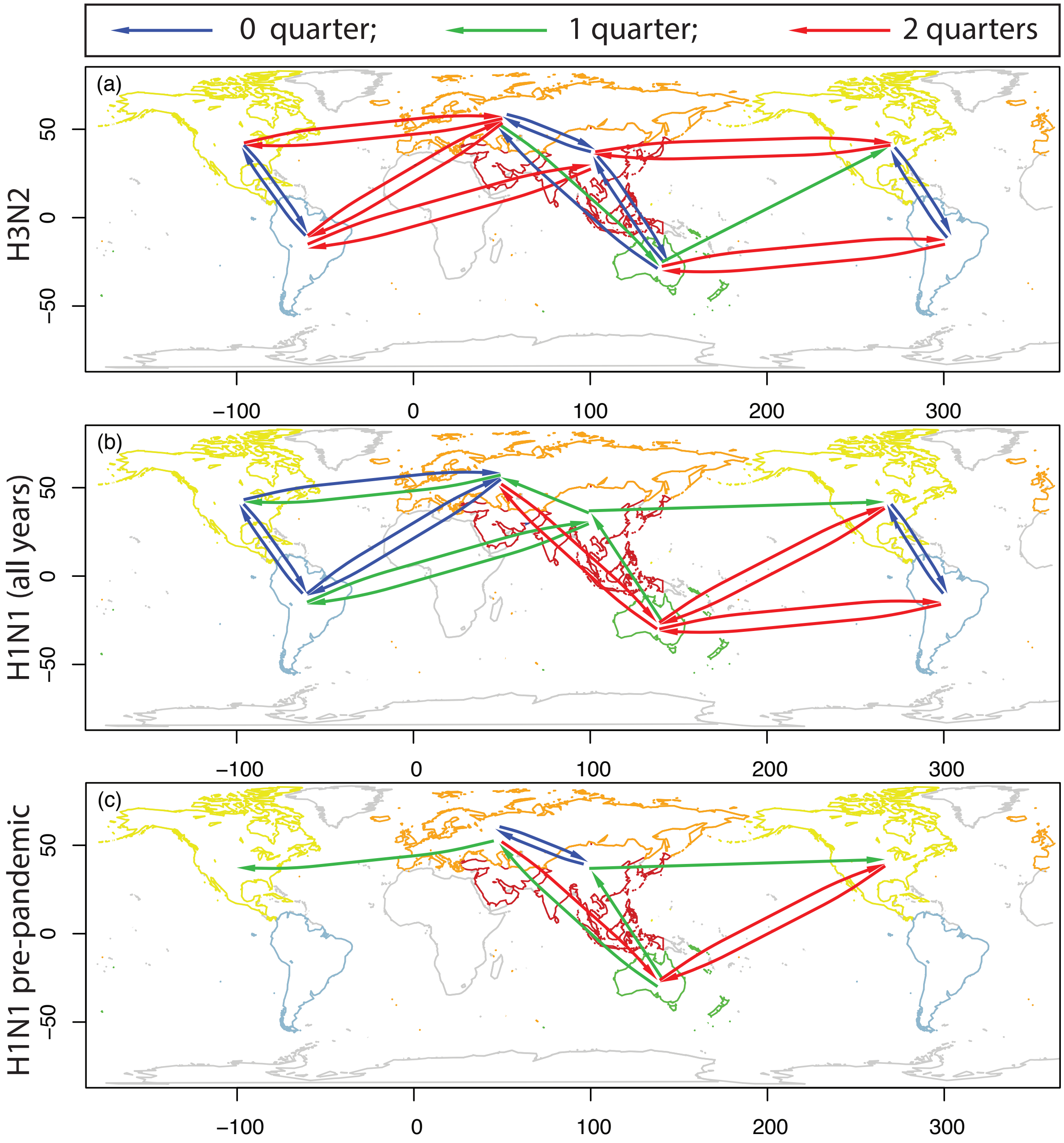

Figure S18. Global absolute connectivity of influenza genetic diversity ν. Networks are shown for: (a) H3N2 viruses (all years), (b) H1N1 viruses (all years) and (c) for pre-pandemic H1N1 viruses (up to Q4/2008). Arrows (directed edges of the graphs) show significant correlations at the 99% level derived from the autocorrelation analysis for lags 0 quarter (blue), 1 quarter (green) and 2 quarters (red).

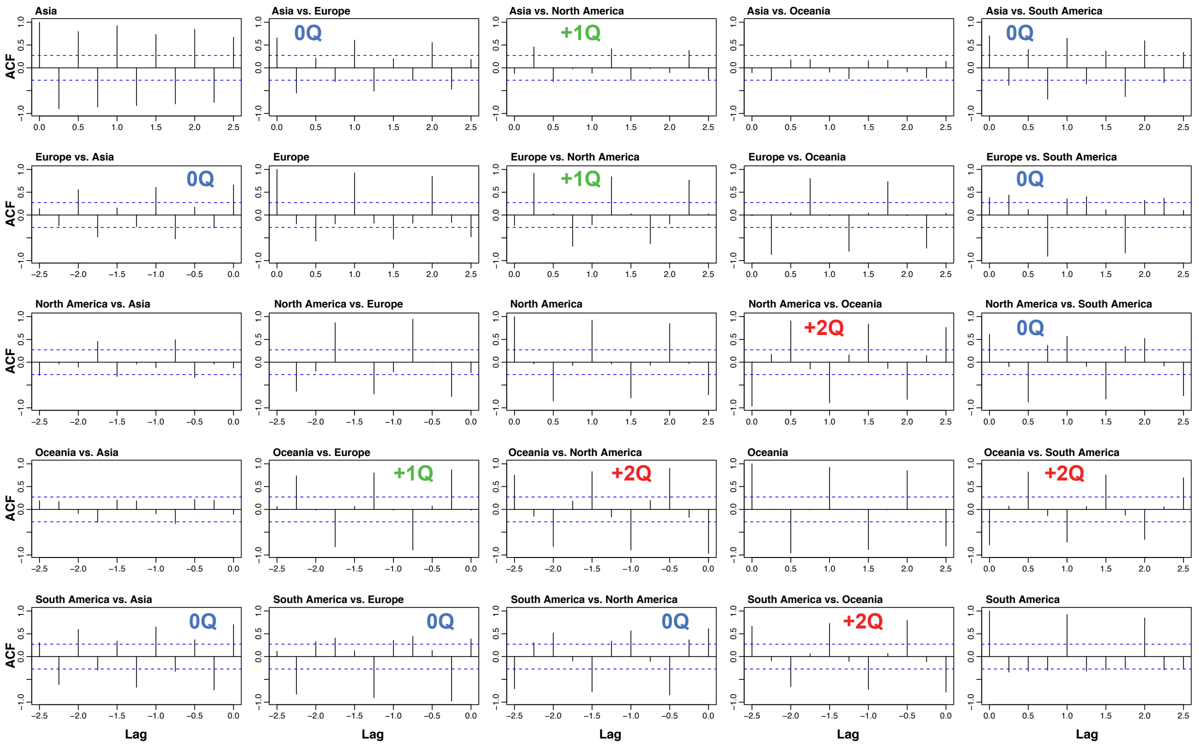

Figure S19. Autocorrelation functions for nucleotide diversity π in pre-pandemic H1 viruses (up to Q4/2008) across the five regions studied here. Regions are ordered as follows: Asia, Europe, North America, Oceania and South America. The time unit for lag is one year. Significant lags are color coded: 0: blue; 1: green; 2: red. Horizontal broken lines: 99% significance level.

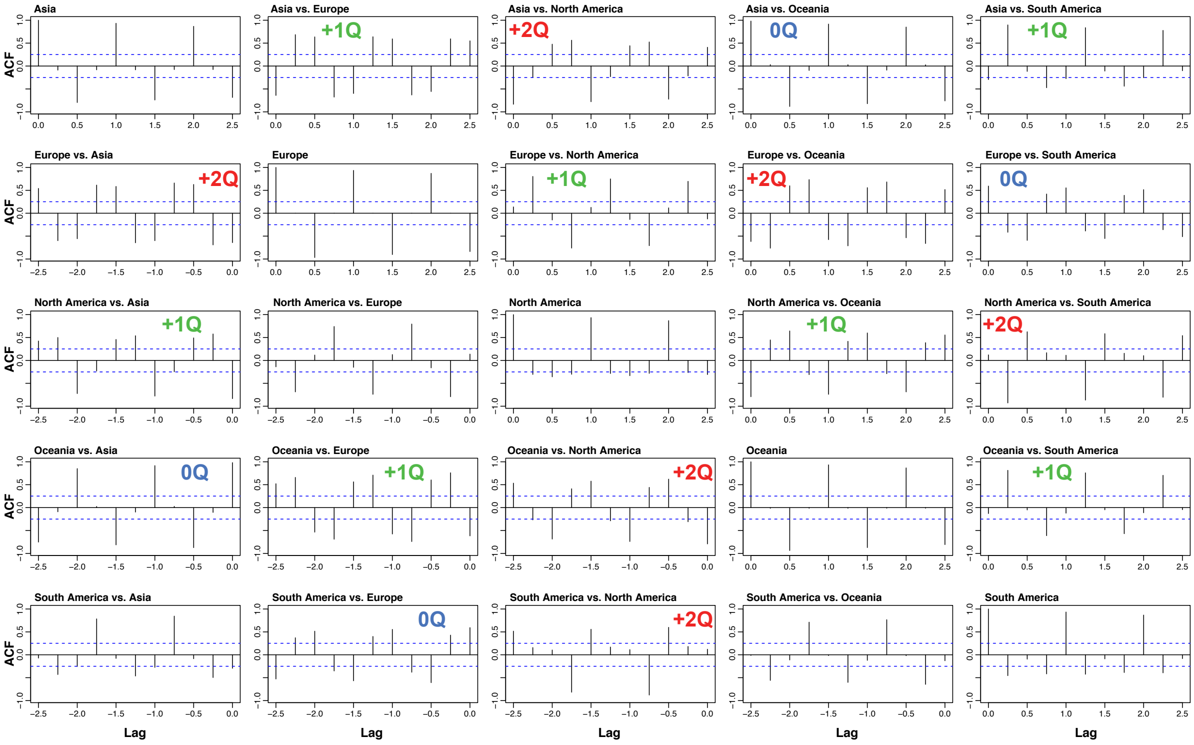

Figure S20. Autocorrelation functions for nucleotide diversity π in H1 viruses across the five regions studied. Regions are ordered as follows: Asia, Europe, North America, Oceania and South America. The time unit for lag is one year. Significant lags are color coded: 0: blue; 1: green; 2: red. Horizontal broken lines: 99% significance level.

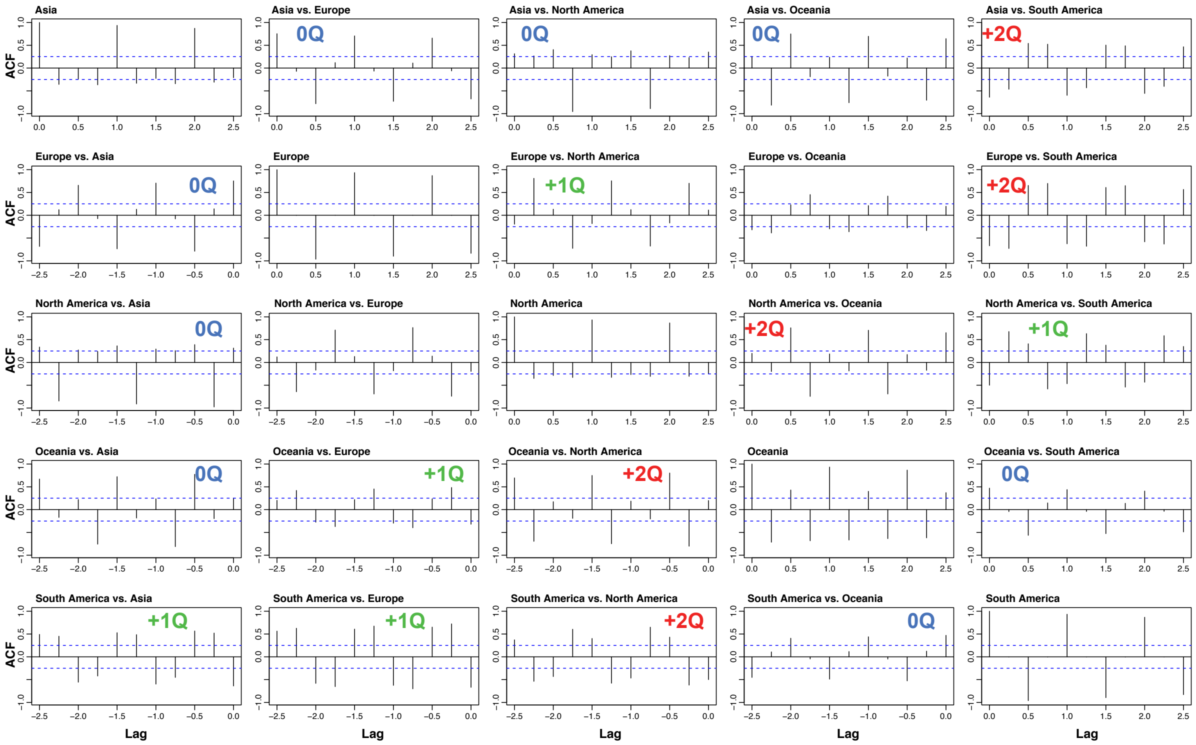

Figure S21. Autocorrelation functions for nucleotide diversity π in H3 viruses across the five regions studied. Regions are ordered as follows: Asia, Europe, North America, Oceania and South America. The time unit for lag is one year. Significant lags are color coded: 0: blue; 1: green; 2: red. Horizontal broken lines: 99% significance level.

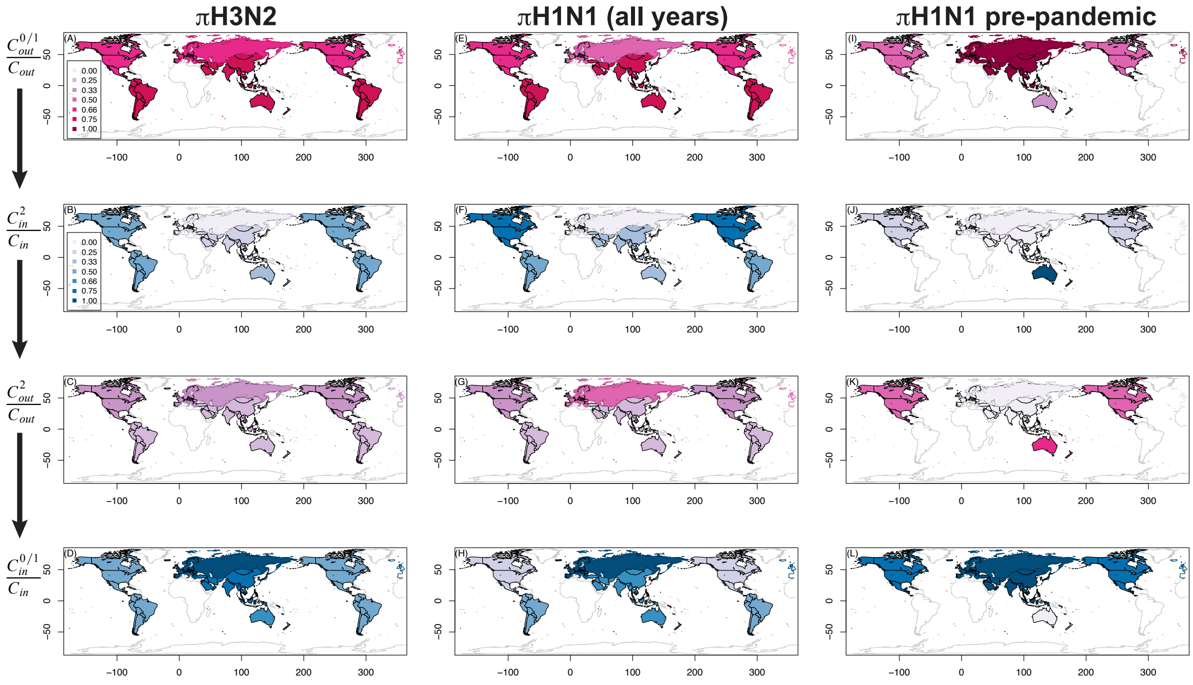

Figure S22. Global relative connectivity of influenza nucleotide diversity π through time. Each column represents the relative connectivity values for H3N2 (A-D), H1N1 (E-H) and pre-pandemic H1N1 (I-L) viruses. Each row represents the temporal connection as described in the text. Relative connectivity values are plotted on two scales: out connectivities are in warm colors, while in connectivities are in cold colors (see insets in panels (A) and (B) for scales).

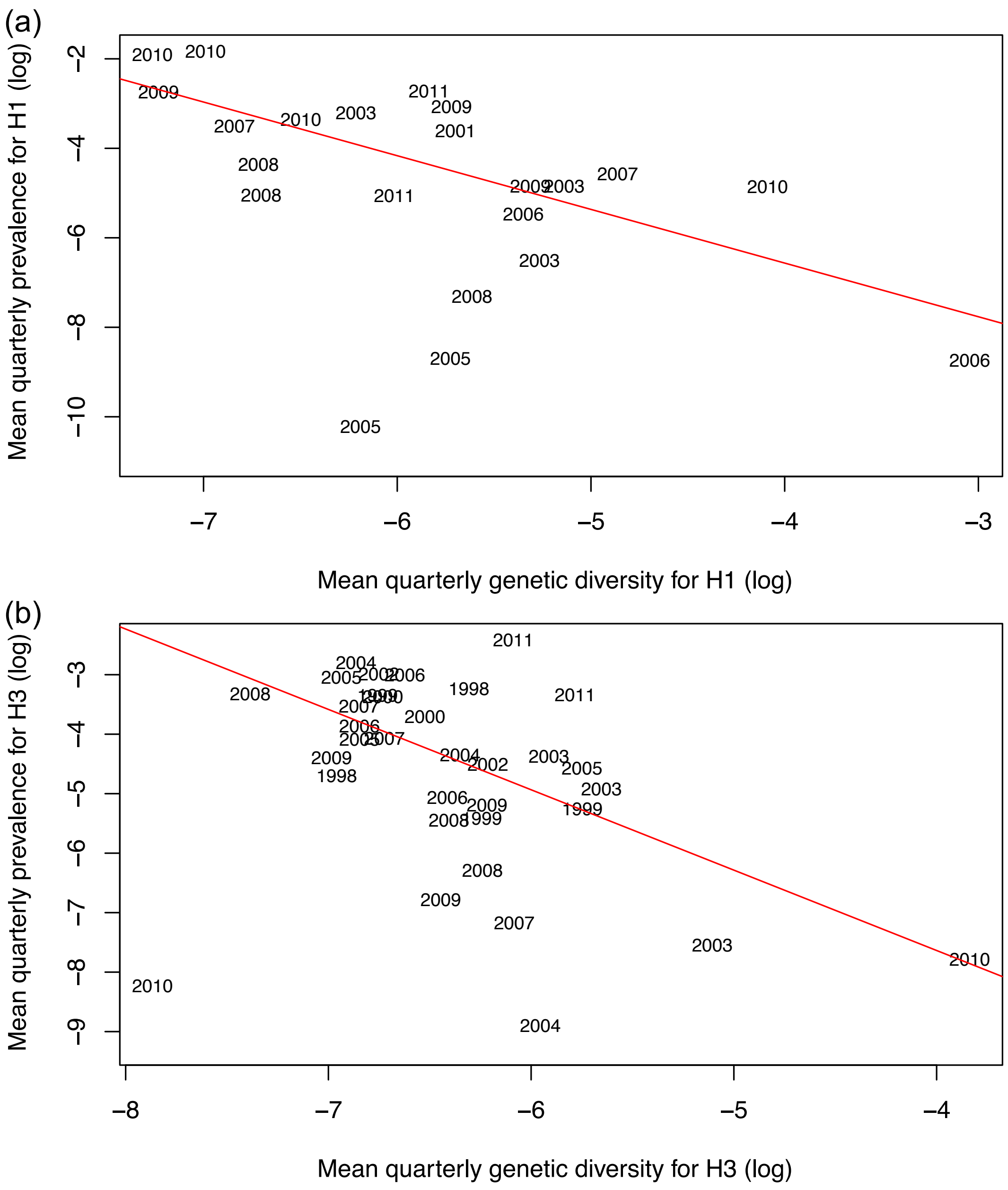

Figure S23. Prevalence as a function of genetic diversity in the US. (a) H1 viruses; (b) H3 viruses. Both panels are on a log-log scale. The red lines correspond to robust regressions for the whole 14.25 years of data.

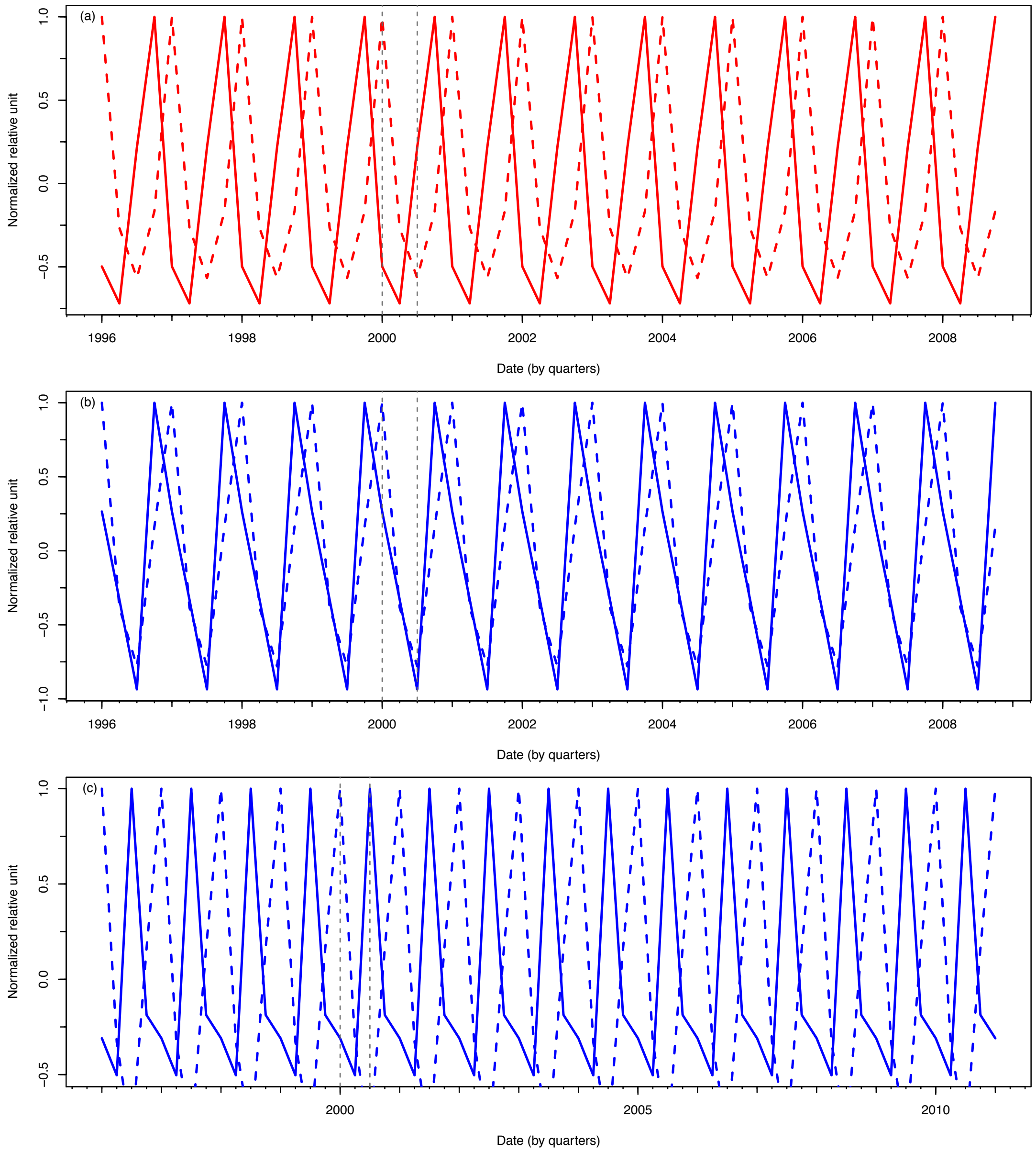

Figure S24. Seasonality of genetic diversity and of prevalence in the US. (a) Pre-pandemic H1 viruses (up to Q4/2008); (b) H3 viruses for the same period; (c) H3 viruses for the complete period. The y-axis is normalized so that both diversity and prevalence take their maximum absolute value at 1. Solid lines: genetic diversity; broken lines: prevalence. Gray vertical lines are drawn at year 2000 and $2000\frac{1}{2}$ for reference.

Supplementary Table

Table S1. Correlation of the timing of ν diversity peaks between regions by subtypes. Correlation coefficients (Pearson's product moment) for H1N1 are above the diagonal and below the diagonal for H3N2. The last line shows the correlation coefficients between H1 and H3 viruses within each region. *P*-values are between brackets. As: Asia; Eu: Europe; Na: North America; Oc: Oceania; Sa: South America.

	As	Oc	Eu	Na	Sa
As	–	0.040 (0.757)	-0.039 (0.765)	0.038 (0.770)	-0.014 (0.912)
Oc	-0.028 (0.832)	–	-0.027 (0.835)	0.114 (0.382)	0.013 (0.922)
Eu	-0.036 (0.781)	0.087 (0.502)	–	0.043 (0.745)	0.032 (0.806)
Na	0.077 (0.553)	0.066 (0.613)	0.017 (0.897)	–	-0.022 (0.866)
Sa	-0.029 (0.825)	-0.037 (0.778)	0.045 (0.731)	0.292 (0.022)	–
H1/H3	-0.052 (0.690)	0.005 (0.967)	-0.064 (0.624)	-0.010 (0.940)	-0.050 (0.702)

Table S2. Connectivity analysis of the five regions in terms of genetic diversity ν. Notations: C_t: total connectivity; C_{out}: distributor connectivity; $C_{out}^{0/1}$: distributor connectivity at lags 0-1; C_{out}^2: distributor connectivity at lag 2; $\frac{C_{out}^{0/1}}{C_{out}}$: contribution as a distributor at lag 0-1; $\frac{C_{out}^2}{C_{out}}$: contribution as a distributor at lag 2. The *in* subscript indicates incoming connectivities. As: Asia; Eu: Europe; Na: North America; Oc: Oceania; Sa: South America; pre-pdm: pre-pandemic; NA: not applicable (no data); *: by convention.

Subtype	Region	C_t	C_{out}	$C_{out}^{0/1}$	C_{out}^2	$\frac{C_{out}^{0/1}}{C_{out}}$	$\frac{C_{out}^2}{C_{out}}$	C_{in}	$C_{in}^{0/1}$	C_{in}^2	$\frac{C_{in}^{0/1}}{C_{in}}$	$\frac{C_{in}^2}{C_{in}}$
H3N2	As	8	4	2	2	0.50	0.50	4	2	2	0.50	0.50
	Eu	7	4	2	2	0.50	0.50	3	2	1	0.67	0.33
	Na	7	3	1	2	0.33	0.67	4	2	2	0.50	0.50
	Oc	7	4	3	1	0.75	0.25	3	2	1	0.67	0.33
	Sa	8	4	1	3	0.25	0.75	4	1	3	0.25	0.75
H1N1 all years	As	5	3	3	0	1.00	0.00	2	2	0	1.00	0.00
	Eu	7	3	2	1	0.67	0.33	4	3	1	0.75	0.25
	Na	7	3	2	1	0.67	0.33	4	3	1	0.75	0.25
	Oc	7	4	1	3	0.25	0.75	3	0	3	0.00	1.00
	Sa	8	4	3	1	0.75	0.25	4	3	1	0.75	0.25
H1N1 pre-pdm	As	4	2	2	0	1.00	0.00	2	2	0	1.00	$0.00^\star$
	Eu	5	3	2	1	0.67	0.33	2	1	0	1.00	0.00
	Na	4	1	0	1	0.00	1.00	3	2	1	0.67	0.33
	Oc	5	3	2	1	0.67	0.33	2	0	2	0.00	1.00
	Sa	NA	NA	NA	NA	NA	NA	NA	NA	NA	NA	NA

Table S3. Connectivity analysis of the five regions in terms of nucleotide diversity π. Notations: C_t: total connectivity; C_{out}: distributor connectivity; $C_{out}^{0/1}$: distributor connectivity at lags 0-1; C_{out}^{2}: distributor connectivity at lag 2; $\frac{C_{out}^{0/1}}{C_{out}}$: contribution as a distributor at lag 0-1; $\frac{C_{out}^{2}}{C_{out}}$: contribution as a distributor at lag 2. The *in* subscript indicates incoming connectivities. As: Asia; Eu: Europe; Na: North America; Oc: Oceania; Sa: South America; pre-pdm: pre-pandemic; NA: not applicable (no data); *: by convention.

Subtype	Region	C_t	C_{out}	$C_{out}^{0/1}$	C_{out}^{2}	$\frac{C_{out}^{0/1}}{C_{out}}$	$\frac{C_{out}^{2}}{C_{out}}$	C_{in}	$C_{in}^{0/1}$	C_{in}^{2}	$\frac{C_{in}^{0/1}}{C_{in}}$	$\frac{C_{in}^{2}}{C_{in}}$
H3N2	As	8	4	3	1	0.75	0.25	4	3	1	0.75	0.25
	Eu	6	3	2	1	0.67	0.33	3	3	0	1.00	0.00
	Na	7	3	2	1	0.67	0.33	4	2	2	0.50	0.50
	Oc	7	4	3	1	0.75	0.25	3	2	1	0.67	0.33
	Sa	8	4	3	1	0.75	0.25	4	2	2	0.50	0.50
H1N1 all years	As	7	4	3	1	0.75	0.25	3	2	1	0.67	0.33
	Eu	7	4	2	2	0.50	0.50	3	3	0	1.00	0.00
	Na	7	3	2	1	0.67	0.33	4	1	3	0.25	0.75
	Oc	7	4	3	1	0.75	0.25	3	2	1	0.67	0.33
	Sa	6	4	3	1	0.75	0.25	2	1	1	0.50	0.50
H1N1 pre-pdm	As	5	3	3	0	1.00	0.00	2	2	0	1.00	$0.00^\star$
	Eu	6	3	3	0	1.00	0.00	3	3	0	1.00	0.00
	Na	6	2	1	1	0.50	0.50	4	3	1	0.75	0.25
	Oc	5	3	1	2	0.33	0.67	2	0	2	0.00	1.00
	Sa	NA	NA	NA	NA	NA	NA	NA	NA	NA	NA	NA
